# Nitric oxide hinders club cell proliferation through Gdpd2 during allergic airway inflammation

**DOI:** 10.1002/2211-5463.13617

**Published:** 2023-05-03

**Authors:** Qing Yue, Kuan Li, Zhaoyu Song, Qi Wang, Jianhai Wang, Xue Li, Yu Li, Qiuyang Zhang, Yu Zhu, Huaiyong Chen

**Affiliations:** ^1^ Department of Basic Medicine, Haihe Clinical School Tianjin Medical University China; ^2^ Department of Basic Medicine, Haihe Hospital Tianjin University China; ^3^ Tianjin Key Laboratory of Lung Regenerative Medicine China; ^4^ Department of Clinical Laboratory, Haihe Hospital Tianjin University China; ^5^ Key Research Laboratory for Infectious Disease Prevention for State Administration of Traditional Chinese Medicine Tianjin Institute of Respiratory Diseases China

**Keywords:** epithelial progenitor cells, glycerin, Ins1p1, lipid metabolism, nitric oxide

## Abstract

Excessive nitric oxide (NO) is often observed in the airways of patients with severe asthma. Here, we show that the NO donor diethylamine NONOate impairs the proliferative capacity of mouse club cells and induces club cell apoptosis, cell cycle arrest, and alterations in lipid metabolism. Our data suggest that NO inhibits club cell proliferation via upregulation of Gdpd2 (glycerophosphodiester phosphodiesterase domain containing 2). During ovalbumin (OVA) challenge, apoptotic club cells are observed, but surviving club cells continue to proliferate. OVA exposure induces Gdpd2 expression; Gdpd2 knockout promotes the proliferation of club cells but inhibits goblet cell differentiation. Elimination of airway NO was found to inhibit goblet cell differentiation from club cells during OVA challenge. Our data reveal that excessive NO might be related to airway epithelial damage in severe asthma and suggest that blockade of the NO‐Gdpd2 pathway may be beneficial for airway epithelial restoration.

## Graphical abstract

Nitric oxide (NO) upregulates Gdpd2 to inhibit club cell proliferation and promotes goblet cell differentiation during OVA‐induced allergic airway inflammation. Persistent excess NO can induce airway epithelial apoptosis and block airway epithelial regeneration. Therefore, targeting the NO‐Gdpd2 pathway may be a potential therapeutic strategy for asthma treatment.

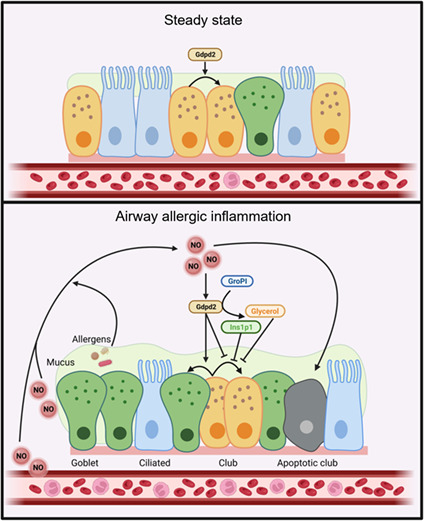

**Abbreviations**
AT1: Alveolar type 1 epithelial cells
AT2: Alveolar type 2 epithelial cells
BALF: Bronchoalveolar lavage fluid
BP: Biological process
CFE: Colony‐forming efficiency
Cyp2f2: Cytochrome P450, family 2, subfamily f, polypeptide 2
DEA NONOate: Diethylamine NONOate
DEGs: Differentially expressed genes
FC: Fold change
FeNO: Fractional exhaled nitric oxide
Gdpd2: Glycerophosphodiester phosphodiesterase domain containing 2
GO: Gene Ontology
Gro3P: Glycerol‐3‐phosphate
GroPI: Glycerophosphoinositol
Ins1p1: Inositol‐1‐phosphate
LNMMA: NG‐monomethyl‐l‐arginine acetate
NES: Normalized enrichment scores
NO: Nitric oxide
OVA: ovalbumin
PBS: Phosphate buffered saline
ppb: Part per billion
qPCR: Quantitative PCR
Scgb1a1: Secretoglobin family member 1a1
scRNA‐Seq: Single‐cell RNA‐sequencing
SFM: Stromal‐free medium
WT: Wild‐type

Nitric oxide (NO) is an inorganic small molecule that can be produced by different cell types, including airway epithelial cells, neurons, endothelial cells, alveolar type 2 epithelial cells, neutrophils, and eosinophils [1]. NO is an essential cellular messenger with roles in the physiological regulation of the cardiovascular, respiratory, and nervous systems [2]. This molecule is involved in signaling and regulation of processes like smooth muscle relaxation, vasodilation, inflammation, and neurotransmission [3, 4]. NO is also a key regulator of physiological functions in the respiratory epithelium, namely epithelial ion transport [5], mucociliary function [6], and barrier restoration after injury [7]. Under healthy conditions, NO is beneficial for the regulation of airway function and maintenance of lung homeostasis. However, excessive NO levels have pathological effects, being associated with increased disease severity in bronchial asthma [8].

Asthma is a chronic respiratory disease characterized by chronic inflammation, airway epithelial damage and remodeling, airway obstruction, and goblet cell metaplasia [9, 10]. The airway epithelium is critical for lung homeostasis and regeneration [11]. Failure of timely repair of the epithelial mucosa after injury is one of the causes for the persistence of inflammation in asthma [10]. Furthermore, both airway epithelial barrier dysfunction and excessive mucus secretion by goblet cells can aggravate asthma development [12, 13]. Club cells, formerly known as Clara cells, are airway epithelial progenitor cells that express cytochrome P450, family 2, subfamily f, polypeptide 2 (Cyp2f2), and secretoglobin family member 1a1 (Scgb1a1) [14]. After epithelial injury, club cells can repair the airway epithelium through self‐proliferation and differentiation into ciliated and goblet cells [15, 16]. Goblet cells are the main mucus‐secreting cells, which are almost nonexistent at a steady state. However, inflammatory stimuli can promote club cell metaplasia into goblet cells, leading to its increase in the airway [17]. Therefore, airway epithelial mucosal damage and goblet cell metaplasia aggravate asthma development. The mechanisms underlying impaired airway epithelial repair and goblet cell metaplasia in asthma, require further studies for a better understanding of the disease.

Under normal physiological conditions, Fractional exhaled nitric oxide (FeNO) is generally lower than 25 ppb (part per billion), but in bronchial asthma, FeNO can exceed 50 ppb, highly indicative of eosinophil inflammation [18]. Lower levels (pM–nM) of NO favor proliferation and survival of bone marrow stromal cells, whereas higher concentrations (μm–mm) promote vascular smooth muscle cell apoptosis and cycle arrest [19]. Nonetheless, the effect of high NO concentration on airway club cells has not yet been reported.

Glycerophosphodiester phosphodiesterase domain containing 2 (GDPD2, also known as GDE3) belongs to a family of six transmembrane proteins, with an external enzymatic domain associated with the bacterial glycerophosphodiester phosphodiesterase [20]. GDPD2 is encoded by *Gdpd2* located on the X chromosome and has been reported to metabolize glycerophosphoinositol (GroPI) to produce inositol‐1‐phosphate (Ins1p1) and glycerol [21]. Gdpd2 is involved in the regulation of cell differentiation, considered a marker of osteoblast differentiation, and negatively regulates cell proliferation [21, 22]. Additionally, Gdpd2 overexpression slowed tumor growth in a mouse xenograft model and inhibited oligodendrocyte precursor cell proliferation via ciliary neurotrophic factor receptor α [23, 24]. However, the role of Gdpd2 in airway epithelial cells has not yet been described.

In this study, to investigate the role of the NO‐Gdpd2 pathway in the development of allergic airway inflammation, we found that NO inhibited club cell proliferation by activating Gdpd2 pathway during ovalbumin (OVA)‐induced allergic airway inflammation and promoted club cell apoptosis. In addition, Gdpd2 promoted the differentiation of club cells to goblet cells in OVA‐challenged mice. These results suggest that targeting the NO‐Gdpd2 pathway may be a potential therapeutic strategy for treating asthma.

## Results

### Nitric oxide inhibited club cell proliferation *in vitro*

**Fig. 1:**
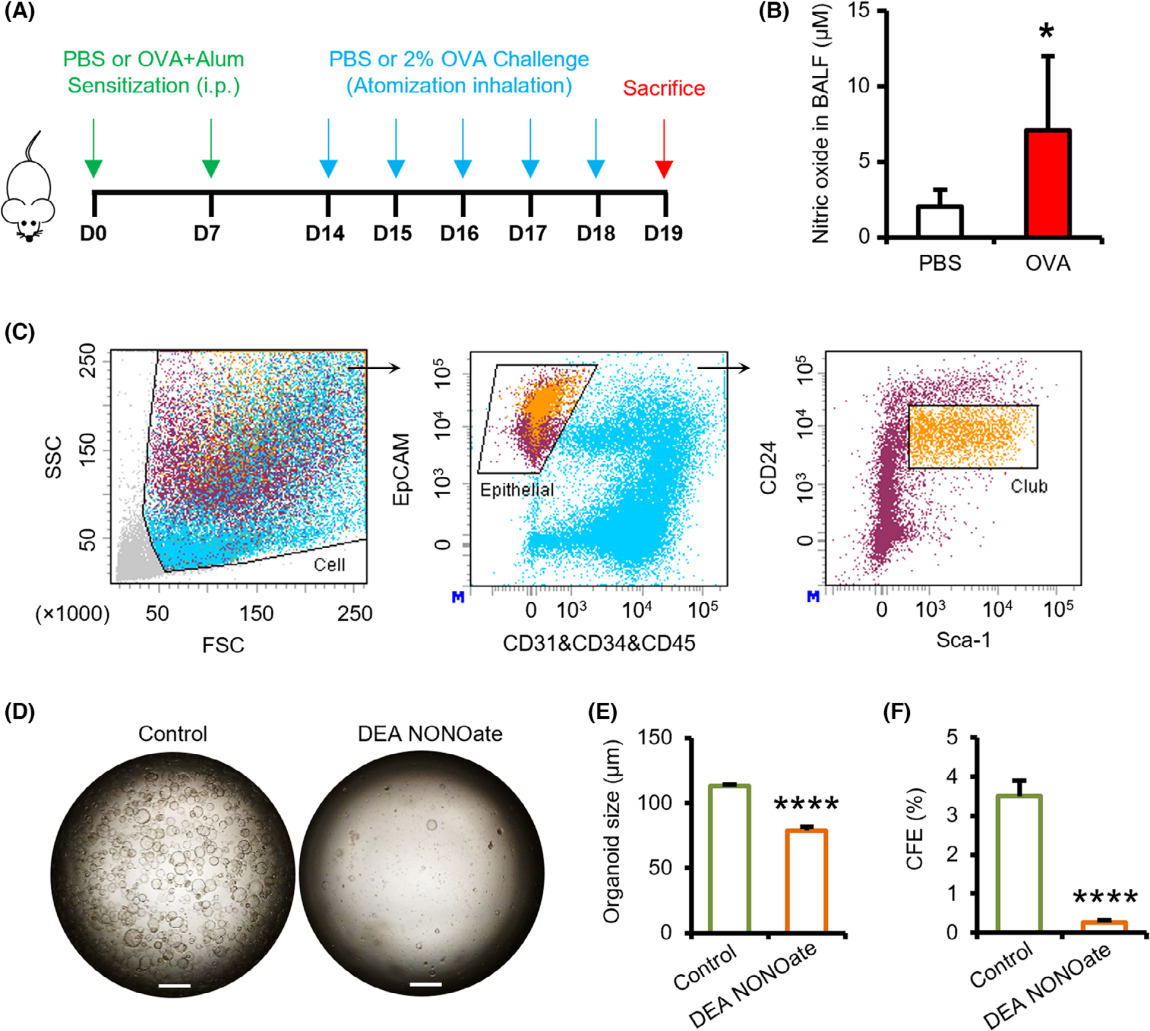
NO inhibits club cell proliferation. (A) Schematic of OVA‐induced airway inflammation model. (B) NO levels in BALF supernatant from PBS and OVA groups (*n* = 5 : 10). (C) Sorting strategy of airway epithelial CD31^−^CD34^−^CD45^−^EpCAM^+^Sca‐1^+^CD24^+^ club cells from naïve mouse lungs, by FACS. (D) Representative images of club cell organoid cultures (stromal‐free system) in the presence of DEA NONOate (25 μm at day 6 after plating (*n* = 5 : 5). Scale bar: 500 μm. (E, F) Colonies diameter (E) and CFEs (F) in club cells from the DEA NONOate group under the conditions described in (D) (*n* = 5 : 5). Results are represented by the mean ± SD, *P < 0.05; ****P < 0.0001; as determined by the Student's *t*‐test.

FeNO level in bronchoalveolar lavage fluid (BALF) of severe asthma can reach as high as 353 ppb, equivalent to 8.8 μm [25]. In our OVA‐induced asthma mouse model (Fig. 1A), NO was found at a concentration of 7.12 ± 4.90 μm in BALF supernatant (Fig. 1B). The NO donor, diethylamine NONOate (DEA NONOate), was used to investigate the effect of NO on club cell function *in vitro*. The concentration of DEA NONOate was set at 25 μm, due to its rapid release kinetics (*t*_*1/2*_ ≈ 2 min) and to mimic the *in vivo* equivalent effect while avoiding an unnecessary overdose, as previously described [26, 27]. EpCAM^+^ CD24^low^Sca‐1^+^ club cells were fractionated from wild‐type (WT) mice by fluorescence activated cell sorting (FACS) for feeder organoid cultures, as previously developed (Fig. 1C) [28, 29]. The average size of club cell‐derived organoids and colony‐forming efficiency (CFE) decreased significantly in DEA NONOate group (Fig. S1A–C). To determine whether the NO‐induced effect on club cells was indirectly mediated via feeder MLg2908 cells, we treated them with DEA NONOate. We observed that NO did not affect MLg cell proliferation or growth factor production (Fig. S1D–G, Table S6). To confirm this, we established feeder‐free organoid cultures by replacing the MLg2908 cells with a set of growth factors and small molecules. Under such conditions, club cells also formed smaller organoids and CFEs in the presence of DEA NONOate compared with the control group (Fig. 1D–F). Therefore, NO directly inhibits club cell proliferation.

### Nitric oxide‐induced club cell cycle arrest and apoptosis

**Fig. 2:**
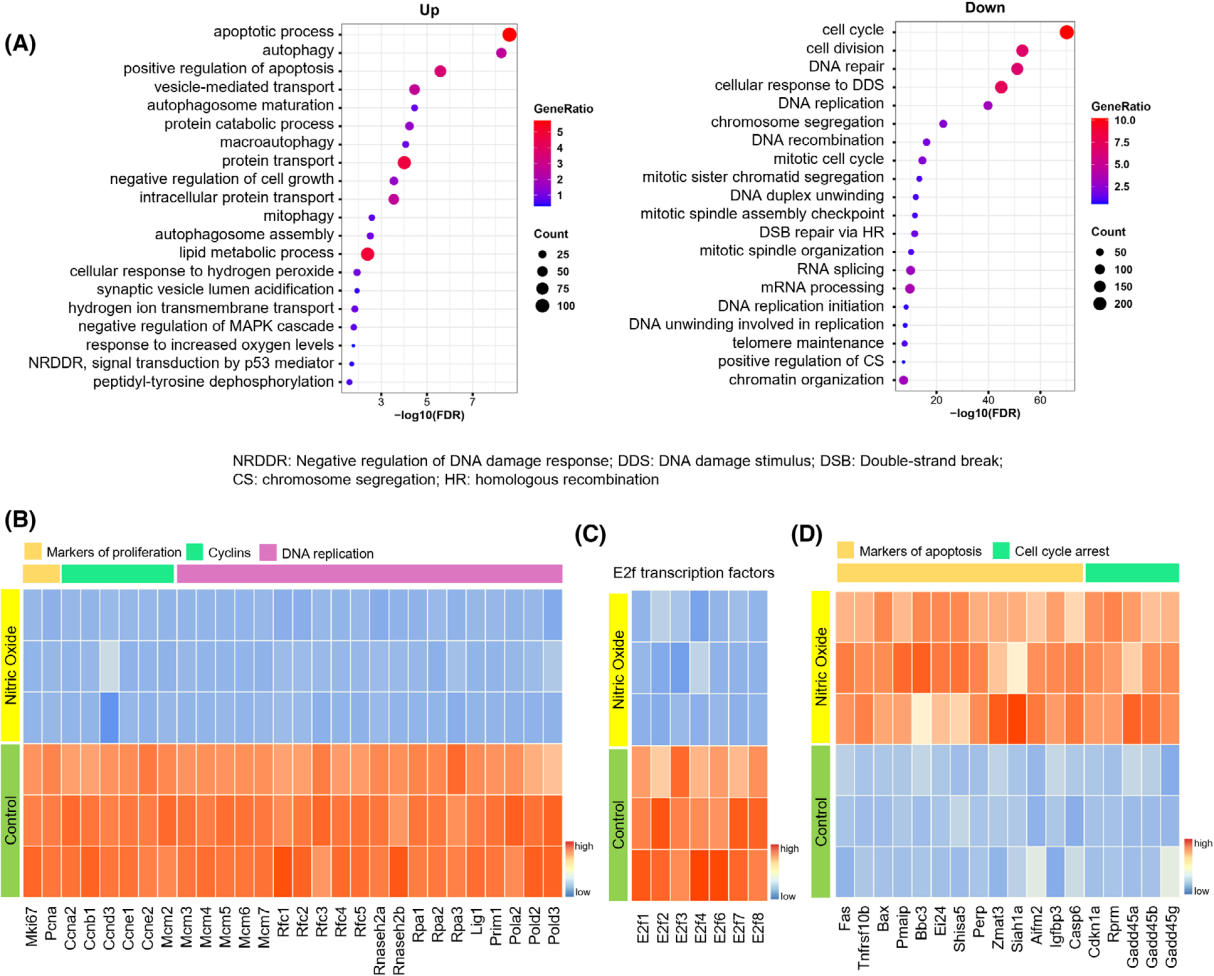
NO induces club cell cycle arrest and apoptosis. (A) Bubble plots showing the top 20 terms (FDR < 0.05) of the GO BP analysis of the upregulated (left) or downregulated (right) DEGs (FC > 1.2, *P*‐adj < 0.05) between NO and control groups, via bulk RNA‐Seq. (B, C) Heatmap depicting the expression of proliferation‐related markers (B) and transcription factors (C) in control and NO groups. FC > 1.2, *P*‐adj < 0.05. (D) Heatmap depicting the expression of apoptosis‐related markers in control and NO groups. FC > 1.2, *P*‐adj < 0.05.

To reveal the underlying mechanisms through which NO inhibits club cell proliferation, bulk RNA‐Seq analysis was performed and 4346 differentially expressed genes [DEGs; NO group vs. Phosphate Buffered Saline (PBS) group] were detected, of which 1910 were upregulated and 2436 downregulated. The biological function of club cells was then evaluated using the biological process (BP) module of Gene Ontology (GO), based on the upregulated and downregulated DEGs. Some of the upregulated DEG‐enriched GO BP terms were associated with stress response, including the ‘apoptotic process’ and ‘mitophagy’ (Fig. 2A) [30, 31]. The downregulated DEG‐enriched GO BP terms were mostly associated with cell proliferation, including ‘cell cycle’, ‘cell division’, and ‘DNA replication’ (Fig. 2A). Genes associated with proliferation, cyclins, and DNA replication were significantly downregulated in the presence of NO (Fig. 2B). More specifically, E2f transcription factor family members, which were enriched in ‘cell circle’ term in Fig. 2A, were significantly downregulated in the presence of NO (Fig. 2C), whereas some downstream targets of the p53 signaling pathway, associated with apoptosis or cell cycle arrest, were significantly upregulated (Fig. 2D). The transcription factors of the DEGs were identified by iRegulon (Fig. S2). Among them, the E2f family, E2f1 and E2f6, were enriched from downregulated DEGs, which had the highest normalized enrichment scores (NES). Together, these data suggest that NO induces club cell apoptosis and cycle arrest *in vitro*.

### *Gdpd2* expression in club cells is upregulated by nitric oxide

**Fig. 3:**
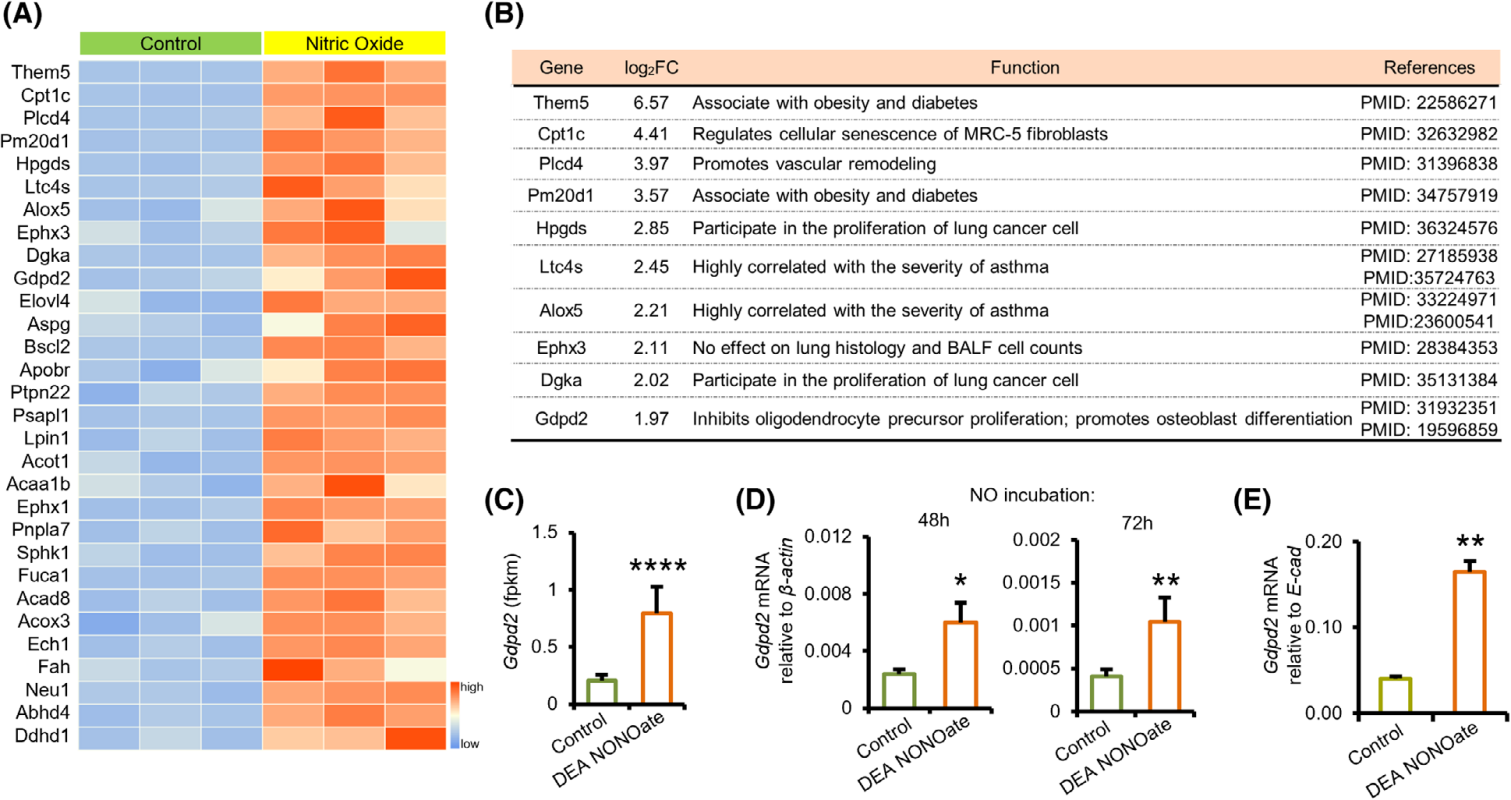
Club cell expression of Gdpd2 is upregulated by NO. (A) Heatmap depicting the expression of the top 30 lipid metabolic markers, in control and NO groups. FC > 1.5, *P*‐adj < 0.05. (B) The functions of top 10 lipid metabolic markers of (A). (C) Bulk RNA‐Seq analysis of Gdpd2 expression in club cells treated with 25 μm DEA NONOate for 24 h. (D) qPCR analysis of Gdpd2 expression (relative to β‐Actin) in club cells treated with 25 μm DEA NONOate for 48 (*n* = 5 : 5) Or 72 (*n* = 5 : 5) Hours. (E) qPCR analysis of Gdpd2 expressions (relative to E‐cad) in organoid cultures (mouse lung fibroblast cell line, MLg system) in the presence of DEA NONOate at day 8 after plating (*n* = 4 : 4). Results are represented by mean ± SD, *P < 0.05; **P < 0.01, ****P < 0.0001; as determined by the Student's *t*‐test.

In addition to ‘apoptosis’, NO upregulated DEGs enriched GO terms also contained the ‘lipid metabolic process’ (Fig. 2A). We analyzed the expression levels of the top 30 genes involved in lipid metabolism in the NO group versus control group (Fig. 3A). The top nine genes have been well characterized as participating in obesity, diabetes, cellular senescence, pulmonary vascular remodeling, asthma severity, and lung cancer cell proliferation (Fig. 3B).

*Gdpd2* expression in club cells was significantly upregulated in the NO group, as detected via bulk RNA‐Seq (Fig. 3C). Quantitative PCR (qPCR) analysis of sorted club cells revealed that *Gdpd2* transcripts were upregulated after incubation with DEA NONOate for 48 or 72 h (Fig. 3D and Table S6). In the feeder organoid model, *Gdpd2* expression was significantly upregulated in the presence of DEA NONOate (Fig. 3E and Table S6). Together, these data suggest NO upregulates *Gdpd2* expression.

### Gdpd2‐catalytic products inhibit club cell proliferation

**Fig. 4:**
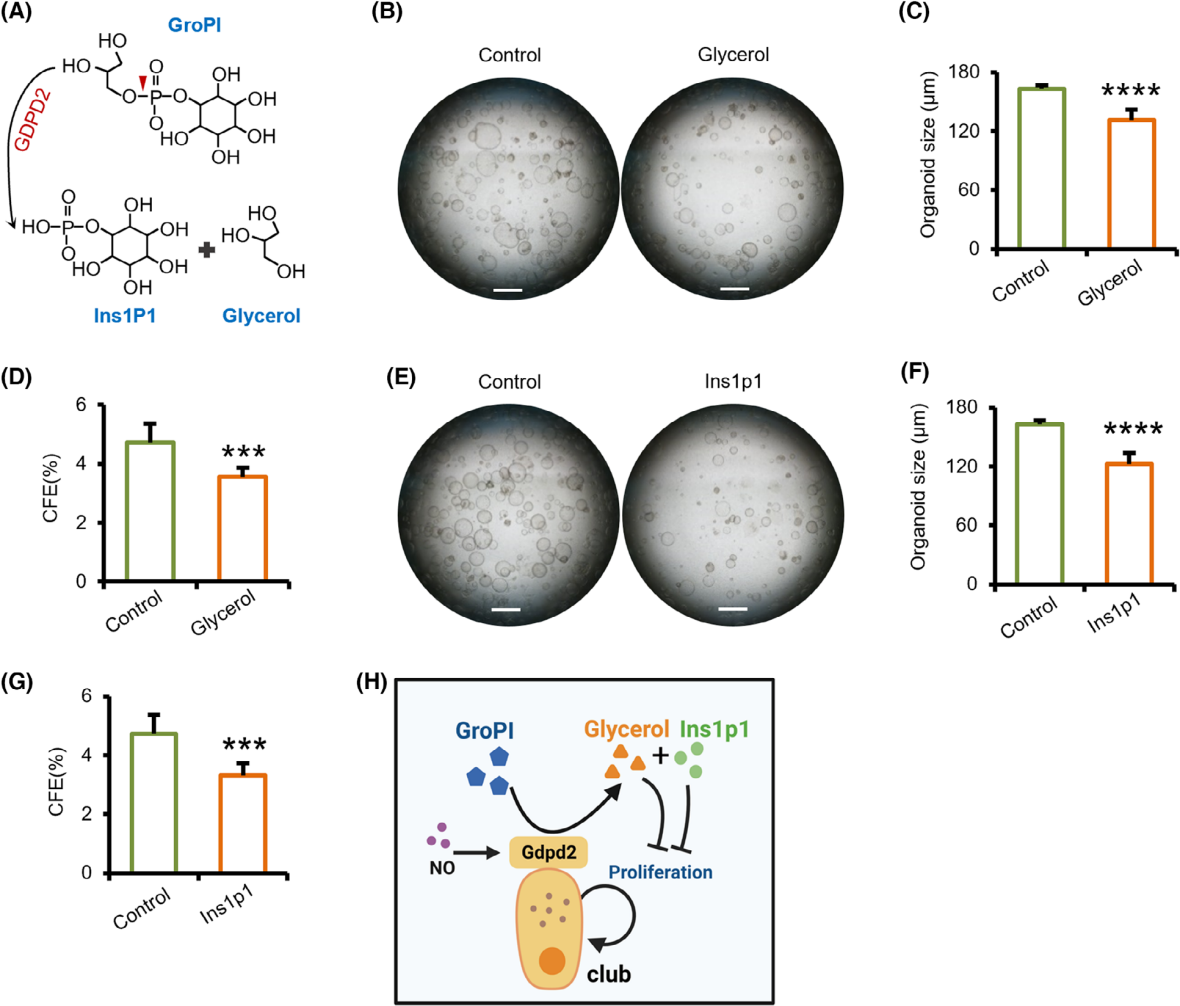
Gdpd2‐catalytic products inhibit club cell proliferation. (A) Gdpd2 catalyzes GroPI to produce glycerol and inositol‐1‐phosphate (Ins1p1). (B, E) Representative images of club cell organoid cultures (stromal‐free system) in the presence of glycerol (100 mm) (*n* = 5 : 5) and Ins1p1 (100 mm) (*n* = 5 : 5) at day 7 after plating. Scale bar: 500 μm. (C, F) Diameter of club cell culture colonies under the conditions described in (B) and (E). (*n* = 5 : 5) (D, G) CFEs of club cell culture colonies under the conditions described in (B) and (E). (*n* = 5 : 5) (H) Schematic illustration of the Gdpd2 catalyzing metabolic pathway. Results are represented by mean ± SD, ***P < 0.001, ****P < 0.0001; as determined by the Student's *t*‐test.

Gdpd2 catalyzes the production of glycerol and Ins1p1 (Fig. 4A) [21]; therefore, we studied the effect of these products on regulating club cell proliferation. Feeder‐free organoid cultures showed reduced organoid size and CFEs of club cell‐derived organoids in the glycerol or Ins1p1 group (Fig. 4B–G). These data suggest that the activation of Gdpd2 metabolic pathway and its catabolites, glycerol and Ins1p1, inhibit club cell proliferation *in vitro* (Fig. 4H).

### Increased club cell apoptosis during OVA‐induced allergic inflammation

**Fig. 5:**
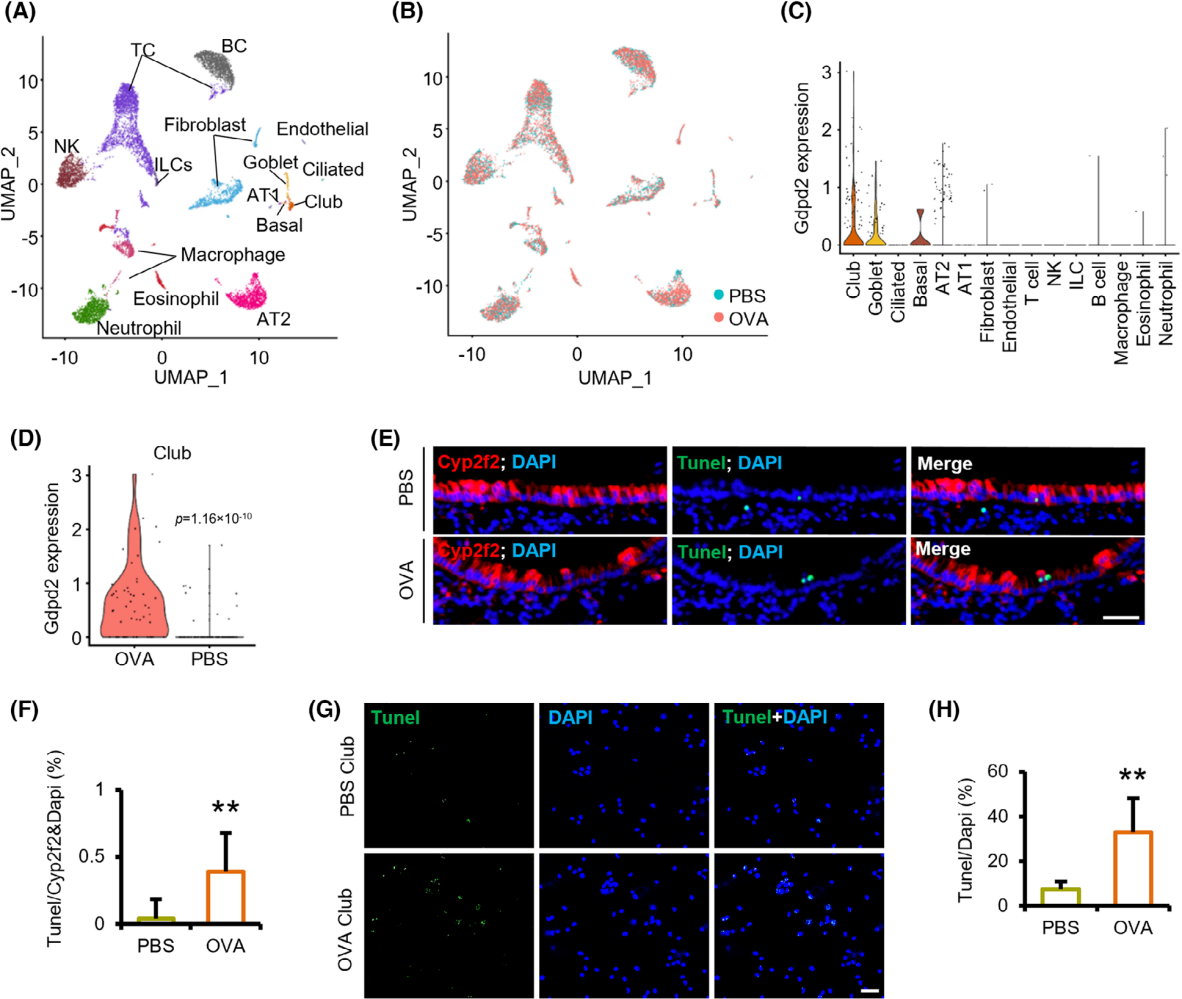
Club cell apoptosis is induced during OVA‐induced allergic inflammation. (A) uMAP plots of 10,217 different cell types. (B) Sample origin of total cells. Blue: PBS group. Red: OVA group. (C) scRNA‐Seq analysis of Gdpd2 expression in each cell type. (D) scRNA‐Seq analysis of Gdpd2 expression in club cells derived from the PBS and OVA groups. (E) Immunofluorescence and TUNEL staining of lungs from PBS or OVA‐challenged WT mice (*n* = 3 : 3). Green: Tunel; Red: CYP2F2; Blue: DAPI. Scale bar: 50 μm. (F) Quantification of TUNEL‐positive cells in the total epithelial cells from the lungs of PBS or OVA‐challenged WT female mice (*n* = 3 : 3). (G) Club cells TUNEL staining (*n* = 6 : 6). Green: TUNEL‐positive cells; Blue: Cell nuclei. Scale bar: 20 μm. (H) Apoptosis rate (TUNEL‐positive) of club cells from the lungs of PBS or OVA‐challenged WT female mice (*n* = 6 : 6). Results are represented by mean ± SD, **P < 0.01, as determined by the Student's *t*‐test.

To test our hypothesis that NO promote club cell apoptosis, we analyzed scRNA‐Seq data of lungs collected from PBS‐ or OVA‐challenged mice, as previously described [32]. After removing batch effects and low‐quality cells, we conducted a standard workflow of the Seurat package for downstream analysis. Natural killer cells, eosinophils, T cells, neutrophils, macrophages, B cells, fibroblasts, innate lymphoid cells, endothelial cells, goblet cells, ciliated cells, basal cells, club cells, alveolar type 1 epithelial cells (AT1), and alveolar type 2 epithelial cells (AT2) were identified (Fig. 5A,B and Fig. S3A). The percentage of club cells decreased while that of airway goblet cells increased in the OVA group (Fig. S3B). Furthermore, Gdpd2 was mainly expressed in club cells and significantly upregulated in the OVA group (Fig. 5C,D).

Proliferative club (Pcna^+^ or Top2b^+^), apoptotic club (Casp2^+^, Casp3^+^, Casp6^+^, Casp7^+^, or Casp8^+^), and quiescent club cells were identified (Fig. S3C–F) and validated by AUCell package and the AddModuleScore function of Seurat package (Fig. S3G). Quiescent club cells were mainly derived from the PBS group, whereas proliferative and apoptotic club cells were mainly derived from the OVA group (Fig. S3D,E). To further characterize the three subtypes during OVA‐induced allergic airway inflammation, we analyzed the cell trajectory (Fig. S4A,B). This revealed club cell development along the quiescent–proliferative/apoptotic–goblet route. The proportion of quiescent clubs in airway cells containing basal, club, goblet, and ciliated cells decreased in the OVA group, while the proportion of proliferative club or apoptotic club cells increased (Fig. S4C). Among the three subsets, Gdpd2 was mainly expressed in proliferative and apoptotic club cells (Fig. S4D) and significantly upregulated in the OVA group (Fig. S4E,F). In addition, we analyzed the genes and pathways that changed along the trajectory. The top 50 genes and the enrichment pathways changed significantly during cell differentiation. (Fig. S4G,H).

Immunofluorescence and TUNEL staining of lung tissue showed an increased percentage of TUNEL‐positive cells in the airways of the OVA group, consistent with the scRNA‐Seq results (Fig. 5E,F). Similarly, the percentage of TUNEL‐positive sorted club cells of the OVA group also increased (Fig. 5G,H). Together, these data suggest that OVA‐induced allergic airway inflammation induces club cell apoptosis while surviving club cells strive to proliferate.

### Gdpd2 reprograms club cell fate after OVA challenge

**Fig. 6:**
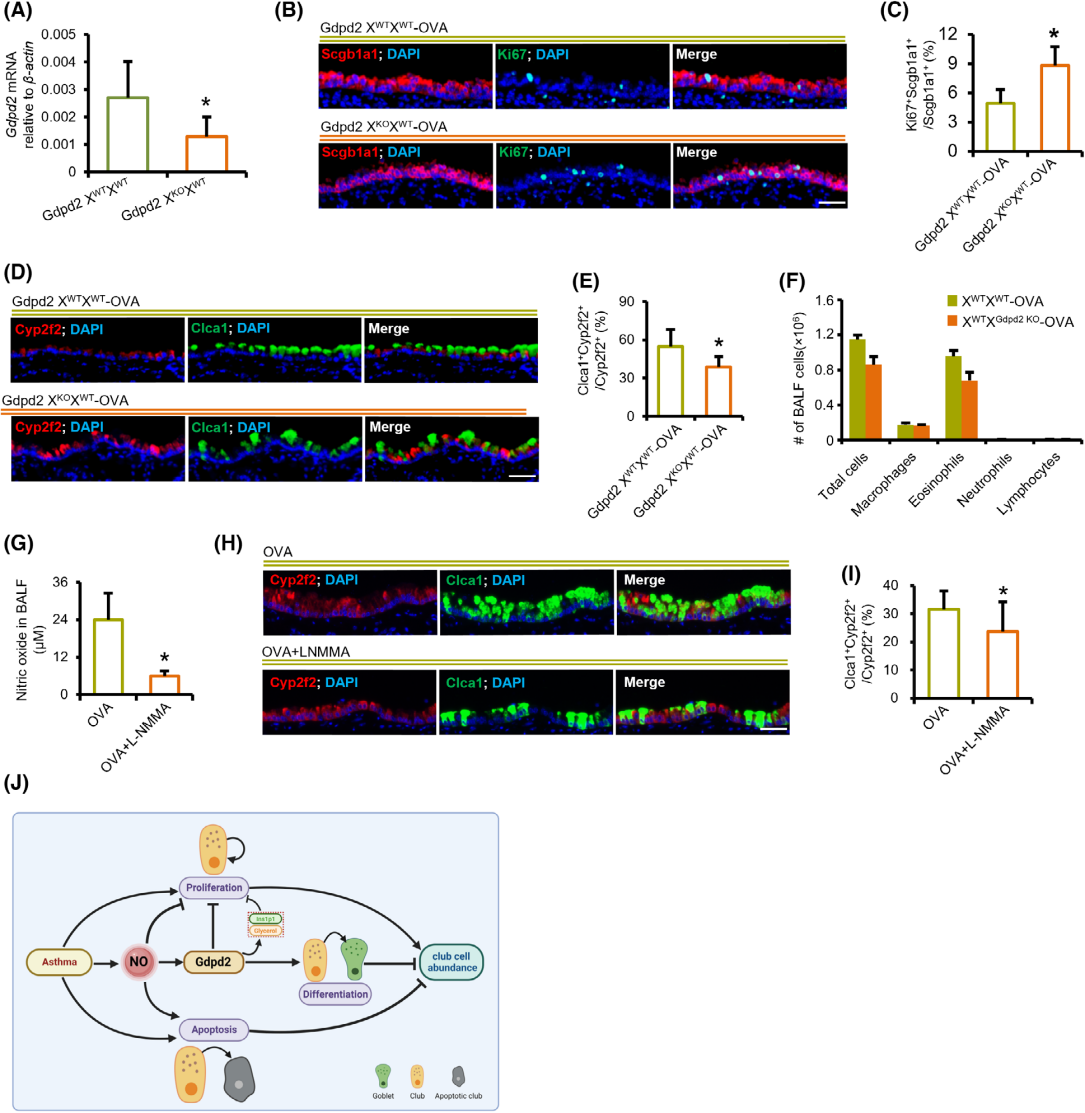
Gdpd2 loss promotes club cell proliferation and reduces goblet cell metaplasia after OVA challenge. (A) qPCR analysis of Gdpd2 expressions (relative to β‐actin) in lungs of WT or Gdpd2 KO (X^KO^X^WT^) female mice (*n* = 4 : 5). (B) Immunofluorescence staining of lungs from OVA‐challenged WT or Gdpd2 KO (X^KO^X^WT^) mice (*n* = 3 : 3). Green: Ki67; Red: Scgb1a1; Blue: DAPI. Scale bar: 50 μm. (C) Quantification of Ki67^+^ cells in the total epithelial cells from the lungs of OVA‐challenged WT and Gdpd2 KO (X^KO^X^WT^) female mice (*n* = 3 : 3). (D) Immunofluorescence staining of lungs from OVA‐challenged WT or Gdpd2 KO (X^KO^X^WT^) mice (*n* = 6 : 6). Green: Clca1; Red: Cyp2f2; Blue: DAPI. Scale bar: 50 μm. (E) Quantification of Clca1^+^ cells in the total epithelial cells from the lungs of OVA‐challenged WT and Gdpd2 KO (X^KO^X^WT^) female mice (*n* = 6 : 6). (F) BALF harvested from the OVA‐challenged WT or OVA‐challenged Gdpd2 KO (X^KO^X^WT^) female mice (*n* = 7 : 7). Infiltrated inflammatory cells were quantified using Hema 3 staining. (G) NO levels in BALF supernatant from OVA and OVA + LNMMA groups (*n* = 4 : 5). (H) Immunofluorescence staining of lungs from OVA or OVA + LNMMA group. Green: Clca1; Red: Cyp2f2; Blue: DAPI. Scale bar: 50 μm. (I) Quantification of Clca1^+^ cells in the total epithelial cells from the lungs of OVA or OVA + LNMMA group (*n* = 8 : 8). (J) Schematic illustration of this research. Results are represented by mean ± SD, *P < 0.05, as determined by the Student's *t*‐test.

To further identify the role of Gdpd2 in club cell proliferation, Gdpd2 knockout (KO) mice were used; the expression was significantly downregulated in the lungs of Gdpd2 heterozygous KO females (X^WT^X^KO^) compared with WT females (X^WT^X^WT^) (Fig. 6A and Table S6). In *in vitro* feeder‐free organoid cultures, the organoid size and CFEs of club cell‐derived organoids from Gdpd2 X^WT^X^KO^ mice were also significantly reduced, compared with those derived from control female mice (Fig. S5A–C). Similar results were observed for Gdpd2 KO male mice (X^KO^Y) (Fig. S5D–F). These data suggest that Gdpd2 is required for club cell maintenance at a steady state.

We further explored the effect of Gdpd2 on club cell fate during OVA‐challenged airway inflammation. Contrary to *in vitro* organoid cultures at steady state, immunofluorescence staining showed that the fraction of Ki67^+^Scgb1a1^+^ was increased over Scgb1a1^+^ club cells in the lung tissues from Gdpd2 X^WT^X^KO^ mice, compared with OVA‐challenged WT female mice (Fig. 6B,C). Club cell differentiation into goblet cells was reduced in the airway epithelium during OVA‐induced allergic inflammation (Fig. 6D,E). However, the quantity of eosinophils, macrophages, and neutrophils in BALF was not significantly different between Gdpd2 X^WT^X^KO^ and WT female mice (Fig. 6F). To verify this, we challenged Gdpd2 X^KO^Y mice with full loss of Gdpd2 and OVA and observed similar results (Fig. S6). Together, Gdpd2 reprograms club cell proliferation and differentiation during OVA‐induced allergic inflammation.

To reveal whether or not dysregulated phenotypes of club cells can be reversed by scavenging NO in asthmatic inflammation, we adopted an inhibitor of NO synthase, NG‐Monomethyl‐l‐arginine Acetate (LNMMA), to reduce the NO level in mouse airways (Fig. 6G). Immunofluorescence staining indicated that reducing airway NO levels inhibited the differentiation of airway club cells into goblet cells in allergic asthmatic mice (Fig. 6H,I).

Altogether, these data suggest that during OVA‐induced allergic airway inflammation, NO activates the apoptotic pathway and inhibits club cell proliferation by activating the Gdpd2‐catalytic pathway. Gdpd2 promotes the differentiation of club cells into goblet cells after OVA treatment. Therefore, the NO‐Gdpd2 pathway disrupts club cell abundance during OVA‐induced allergic inflammation (Fig. 6J).

## Discussion

Excess NO was observed in the BALF of asthmatic and OVA‐induced allergic airway mice. In this study, using *in vitro* 3D organoid model, we found that NO acts directly on club cells, inhibiting their proliferation. Furthermore, bulk RNA sequencing further supported that NO might inhibit club cell proliferation, and also promote apoptosis. Additionally, NO promotes Gdpd2 expression in mouse club cells and its catalytic products, namely glycerol and Ins1p1, can inhibit club cell proliferation. Using Gdpd2 KO mice, we observed an inhibition of club cell proliferation at a steady state, with a significant promotion of club cell proliferation and reduced goblet cell metaplasia, during OVA‐induced allergic airway inflammation. Reducing airway NO levels may inhibit the differentiation of club cells into goblet cells in allergic asthmatic mice.

At the physiological level, NO is an important signaling molecule for homeostasis and is essential for normal tissue function, regulating pulmonary vasodilation, bronchodilation, ciliary beat frequency, host defense, and allergic inflammation [33, 34, 35]. NO is produced by several cell types, such as airway epithelial cells, endothelial cells, neurons, alveolar epithelial type 2 cells, neutrophils, and eosinophils, the latter being considered a marker of airway inflammation [1]. However, excess NO levels may aggravate airway obstruction in patients with asthma [2]. In fact, there is a positive correlation between the exhaled NO and airway eosinophilic inflammation [34]. We previously demonstrated that eosinophils impair club cell proliferation [32] and excess NO has been shown to promote muscle cell apoptosis and cycle arrest [19]. In our OVA‐challenged mouse model, NO concentration in the BALF was equivalent to that observed in patients with severe asthma [19]. Furthermore, we revealed that 25 μm DEA NONOate could impair club cell proliferation in the mouse airway. The migration of newly generated lung epithelial cells is necessary for the restoration of the airway epithelia after injury. An *in vitro* migration assay suggested that human bronchial epithelial cell migration was impaired upon exposure to 500 μm DEA NONOate [36]. Exposure to this concentration of this NO donor can disrupt the distribution of several key tight junction proteins in human bronchial epithelial cells [7]. However, such effects were not observed when the cells were exposed to 100 μm DEA NONOate. Therefore, NO influences epithelial regeneration, migration, and barrier reorganization in the airway.

Cell survival, growth, proliferation, and differentiation depend on cell metabolism. Cell proliferation requires various biological macromolecules, such as proteins and lipids, to produce new cells [37]. We previously observed that glucose deprivation and glycolysis blockade abrogated the proliferative potential of airway club cells while promoting ciliated and goblet cell differentiation [29]. Glucose uptake regulation by autophagy has been demonstrated previously [29, 38]. Here, we found that autophagy was induced in the surviving club cells exposed to excess NO (Fig. 2A). These data suggest that when the proliferative potential of club cells is suppressed by NO during OVA‐induced allergic inflammation, these cells manage stress signals to proliferate. NO‐mediated metabolic alterations include altered lipid metabolic processes in club cells. We found OVA‐induced and NO‐induced upregulation of Gdpd2, a protein catalyzing GroPI to produce glycerol and Ins1p1, which are subclasses of lipid metabolites, in club cells. Vegetable glycerin e‐cigarette aerosols cause inflammation and mucus hyperconcentration, which may promote airway injury [39, 40]. Glycerol, at 2–4%, significantly inhibits cell proliferation in carcinoma cells [41, 42]. In this study, glycerol and Ins1p1 also inhibited club cell proliferation *in vitro*, indicating that these lipid metabolites may be downstream NO mediators, which inhibit club cell proliferation by upregulating Gdpd2 expression.

Nonetheless, the role of Gdpd2 in club cell proliferation has shown some contrasting results. Gdpd2 KO mice exhibited increased club cell proliferation and reduced goblet differentiation during OVA‐induced allergic inflammation. By contrast, Gdpd2 decrease *in vitro*, in organoid models, inhibits club cell proliferation. This clearly indicates that a niche environment is essential for redirecting club cell regulation by Gdpd2. The direct Gdpd2‐catalytic products, glycerol and Ins1p1, might generate different metabolites via various pathways that differentially regulate club cell functions at steady state versus in allergic airway inflammation. As a regulator of lipid metabolism, Lkb1 regulates club cell proliferation in a niche‐dependent manner [43, 44]. Glycerol inhibits cell proliferation but may promote cell proliferation under some pathological conditions. AQP3 promotes epidermal proliferation and skin cancerization by transporting glycerol [45]. Additionally, glycerol participates in multiple lipid metabolic pathways. Glycerol can participate in phosphorylation events in AT2 cells [46]. Glycerol can be catalyzed to produce glycerol‐3‐phosphate (Gro3P) by dephosphorization of Gro3P phosphatase or by hydrolysis of glycerol kinase [47]. The biosynthesis of Gro3P can replenish NAD^+^ to restore partial mitochondrial function, enhancing the proliferation of mitochondria‐damaged cells [48]. Therefore, glycerol may exert different functions under various pathological conditions. However, there is little research on club cells or other types of lung tissue cells favor the glycerol metabolism pathway under homeostasis and acute inflammation. It is likely that there is a glycerol metabolic transition from the pro‐proliferation pathway to the anti‐proliferation pathway when homeostasis shifts toward acute inflammation. Further studies are necessary to determine the mechanisms underlying the opposing Gdpd2 function in an inflammatory environment and homeostasis.

In conclusion, the present results revealed that NO upregulates Gdpd2 to inhibit club cell proliferation and promote goblet cell differentiation during OVA‐induced allergic airway inflammation. Persistent excess NO can induce airway epithelial apoptosis and block airway epithelial regeneration. Therefore, targeting the NO‐Gdpd2 pathway may be a potential therapeutic strategy for asthma treatment.

## Materials and methods

### Mice

C57BL/6 mice and Gdpd2 KO mice were obtained from Sipeifu Biotechnology Co., Ltd. (Beijing, China) and GemPharmatechTM (Nanjing, China), respectively. The mice required for the experiment were housed at the animal facility of Tianjin Haihe Hospital (Tianjin, China) under specific pathogen‐free conditions. All mouse experiments strictly comply with the guidelines approved by the Institutional Animal Care and Use Committee of Tianjin Haihe Hospital (2022HHSQKT(A)‐005).

### Cell lines

MLg2908 (the mouse lung fibroblast cell line) was purchased from ATCC (Manassas, VA, USA) and cultured as described previously [32].

### Lung dissociation and flow cytometry

Single‐cell suspension was obtained as described previously [32]. Briefly, whole lungs of mice were hydrolyzed with elastase and the tissues were cut into pieces. Next, tissue homogenate was hydrolyzed with DNase I (Sigma‐Aldrich). A 70‐μm cell strainer (Falcon; BD Biosciences, San Jose, CA) was used to filter the single‐cell suspension. After removing potential interferences, the cell suspensions were incubated with antibodies (Table S1). The sorted mouse club cells (EpCAM^+^CD24^low^Sca‐1^+^) were resuspended in the culture medium (Table S2 and S3) for subsequent experiments.

### Organoid cultures

Mouse club cells (3 × 10^3^/per well) were sorted and co‐cultured with MLg fibroblasts (2 × 10^5^/per well) as described previously [32]. On days 8 or 10 after seeding, the colony size was evaluated using a DP80 inverted fluorescence microscope (Olympus, Tokyo, Japan). As indicated, stromal‐free organoid cultures were developed with sorted mouse club cells (5 × 10^3^/per well) as described previously [49]. Briefly, the process of club cell seeding was the same as that for organoid co‐culture with MLg, in which the basic culture medium was replaced with a stromal‐free medium (SFM). The composition of SFM is described in Table S3.

### RNA extraction and qPCR

According to the manufacturer's protocol, total RNA was extracted from mouse lung tissue, club cell‐derived organoids, and isolated airway club cells with Trizol reagent (Invitrogen, Carlsbad, CA, USA). qPCR analysis was performed as described previously [32].

### Immunofluorescence staining

Mouse lung tissues were fixed, dehydrated, embedded, sectioned, and immunofluorescence stained as described previously (Table S4 and S5) [32]. A DP80 inverted fluorescence microscope (Olympus) was used to observe the stained tissues. The DeadEnd Fluorimetric TUNEL System (Promega, Madison, WI, USA) was used for TUNEL staining according to the manufacturer's protocol.

### Ovalbumin–induced allergic lung inflammation

C57BL/6 and Gdpd2 KO mice were used for the implementation of the OVA‐induced asthma models [29, 32]. Briefly, all animals were randomly assigned into two groups, PBS and OVA. Mice were sensitized on days 0 and 7 and then were challenged with 2% OVA diluted in PBS by ultrasonic atomization inhalation, for 30 min each day on days 14 to 18. Lung tissues were collected on day 19.

### Bronchoalveolar lavage fluid processing

Bronchoalveolar lavage fluid was obtained and stained for cell type identification [29, 32]. NO assay kit (Nanjing jiancheng Bioengineering Institute, Nanjing, China) was performed to detect the NO level during OVA‐induced airway inflammation according to the manufacturer's protocol. Briefly, the cells were resuspended in 40 μL of PBS and spread on slides, and stained with Hema 3™ (Fisherbrand, Pittsburgh, PA, USA) to characterize eosinophils, macrophages, neutrophils, and lymphocytes.

### Club cell treatment with DEA NONOate

Club cells were separated with flow cytometry and resuspended in SFM with or without 25 μm DEA NONOate (Cayman chemical company, Ann Arbor, MI, USA). A total of 1.9 × 10^5^ cells was then added to each well of the 96‐well plate and incubated at 37 °C with 5% CO_2_ for 48/72 h.

### Bulk RNA‐Seq

Total RNA was extracted from control (*n* = 3) and DEA NONOate‐treated club cells (*n* = 3; 1.9 × 10^5^ cells/sample) using TRIzol reagent (Invitrogen). The club cells were separated with flow cytometry from C57BL/6 mice and treated with or without DEA NONOate for 24 h. Bulk RNA‐seq library construction, sequencing, and data processing were performed as described previously [32]. We used DESeq2 to identify the DEGs. Genes with an adjusted *P*‐value < 0.05 and |FC| > 1.2 (FC: fold change) were considered significant. GO BP analysis of differential expression genes was performed by the DAVID (updated on December 22, 2022, https://david.ncifcrf.gov/). The heatmaps were plotted using pheatmap function of pheatmap package. The features × cells matrices data were inputted and then scaled by setting the scale parameter to the feature dimension (column). The processed data were ranging from −1 (blue) to 1 (red), which represented the lowest and highest values, respectively. Main enriched transcription factors of DEGs were performed by iRegulon, a plug‐in of the Cytoscape software.

### Single‐cell RNA‐Seq

The single‐cell RNA‐sequencing (scRNA‐Seq) dataset of lung tissues from untreated (*n* = 1) or OVA‐induced asthmatic mice (*n* = 1) we previously developed were reanalyzed (accession number: GSE203079). scRNA‐Seq data processing was performed as described previously [32]. For the PBS group, cells with more than 5500 and fewer than 200 genes or those composed of more than 30% UMI related to mitochondrial genome or over 5% UMI related to hemoglobin were considered low‐quality cells and were removed. For the OVA group, cells with more than 4500 and fewer than 200 genes or those composed of more than 20% UMI related to mitochondrial genome or over 5% UMI related to hemoglobin were removed. After filtering, the Seurat package was used for downstream analysis. ‘LogNormalize’ was used to normalize the data and ‘FindVariableFeatures’ was used to calculate the highly variable genes. Canonical marker genes were used to annotate the primary and fine cell types in the dataset. The subtypes of club cells were verified through the ‘AUCell’ package and ‘AddModuleScore’ function of Seurat package using markers of proliferation and apoptosis.

### Pseudotime analysis

After the clustering of the club cells, we conducted a cell differentiation trajectory analysis using R package ‘monocle 2’ [50]. The count data and cell metadata were first submitted individually to create a CellDataSet object using a negative binomial distribution with a fixed variance method. Then, the genes expressed in fewer than 5 cells were removed from the object, and the rest of the genes were used for calculating the pseudotime trajectory. Cell trajectory was constructed by Discriminative Dimensionality Reduction with Trees algorithm. Furthermore, the top 50 genes with the lowest q‐value that changed along the cell differentiation trajectory were retained to perform pathway enrichment analysis via the DAVID.

### Statistical analysis

All experimental data were displayed as mean ± standard deviation. Differences between the experimental and control group were analyzed by the Student's *t*‐test. To compare the proliferative and apoptotic scores of club cells, we used the two‐sided Wilcoxon rank‐sum test by wilcox.test function of the stats package. *P*‐values <0.05 were considered to be statistically significant (*P < 0.05, **P < 0.01, ***P < 0.001).

## Conflict of interest

The authors declare no conflict of interest.

### Peer review

The peer review history for this article is available at https://www.webofscience.com/api/gateway/wos/peer‐review/10.1002/2211‐5463.13617.

## Author contributions

HC involved in conceptualization; QY, KL, ZS, QW, JW, XL, YL, and QZ involved in investigation; QY, KL, and ZS involved in formal analysis; KL and QY involved in data curation; QY, KL, and HC involved in writing—original draft preparation; KL and HC involved in writing—review and editing; HC and YZ involved in supervision; HC and YZ involved in project administration. All authors have read and agreed to the published version of the manuscript.

## Supporting information


**Fig. S1.** Nitric oxide inhibits club cell proliferation in stromal‐feed organoid cultures. (A) Representative images of club cell organoid cultures (co‐cultured with mouse lung fibroblast cell line (MLg)) in the presence of 25 μM diethylamine NONOate (DEA NONOate) at day 8 after plating (n = 5:5). Scale bar: 500 μm. (B, C) Diameter and CFEs of club cell colonies from the DEA NONOate group under the conditions described in (A) (n = 5:5). (D) Numbers of MLg in control and nitric oxide (NO)‐treated groups (n = 6:6). (E, F, G) qPCR analysis of Fgf7 (E), Fgf10 (F), and Hgf (G) expressions (relative to β‐actin) of MLg, cultured in a 100‐mm petri dish for 72 h in the presence of DEA NONOate (25 μM) (n = 6:6). Results are represented by mean ± SD, ****p < 0.0001; as determined by Student's t‐test.

**Fig. S2.** Enrichment analysis of transcription factor from bulk RNA‐seq. Main enriched transcription factors of the differentially expressed genes identified by iRegulon. The normalized enrichment scores (NES) and the number of target genes are indicated.

**Fig. S3.** Identification of club subsets during OVA‐induced allergic inflammation. (A) Dot map depicting specific marker genes for each cell subtype. (B) scRNA‐Seq analysis of club and goblet cells percentage in total airway cells. Airway cells consist of club cell, goblet cell, basal cell and ciliated cell. (C) Specific marker genes for proliferative, apoptotic, and quiescent club cells, and goblet cells. (D) uMAP plots of club cells subsets and goblet cells. (E) Sample origin of club and goblet cells. Blue: PBS group. Red: ovalbumin (OVA) group. (F) Expression of specific marker genes of proliferative club, apoptotic club, quiescent club, and goblet cells. (G) Plots of proliferative and apoptotic scores (AUCell score and Module score) for three club cell subtypes using corresponding cell type markers. The AUCell scores are represented by mean ± SD; The Module scores are represented by median. Differences among the groups were analyzed by the two‐sided Wilcoxon rank‐sum test by wilcox.test function of the stats package.

**Fig. S4.** Altered subsets of club cells during OVA‐induced allergic inflammation. (A) Pseudotime developmental trajectory analysis from Monocle2 depicting relations between proliferative club, apoptotic club, quiescent club, and goblet cells. (B) Pseudotime labelling of goblet cells and three club cells subsets. (C) Club cells subtypes percentage in total airway cells. (D) Single‐cell RNA seq analysis of Gdpd2 expression in each club cells subset. (E, F) Single‐cell RNA seq analysis of Gdpd2 expression in apoptotic (E) or proliferative (F) club cells. Differences were analyzed by the two‐sided Wilcoxon rank‐sum test based on the FindMarkers function of the Seurat package, p < 0.05 were considered to be statistically significant. (G) Expression heatmap showing top 50 genes with the lowest q value along the cell differentiation. (H) Top 20 pathway enrichment results of genes altered in differentiation process.

**Fig. S5.** Gdpd2 deficiency inhibits club cell proliferation *in vitro*. (A) Representative images of club cell organoid cultures (stromal‐free system) from wide‐type mice or Gdpd2 KO (X^KO^X^WT^) mice (n = 5:5), at day 8 after plating. Scale bar: 500 μm. (B, C) Diameter and CFEs of club cell colonies under the conditions described in (A) (n = 5:5). (D) Representative images of club cell organoid cultures (stromal‐free system) from wide‐type mice or Gdpd2 KO (X^KO^Y) mice (n = 5:5), at day 8 after plating. Scale bar: 500 μm. (E, F) Diameter and CFEs of club cell colonies under the conditions described in (C) (n = 5:5). Results are represented by mean ± SD, *p < 0.05, ***p < 0.001, ****p < 0.0001; as determined by Student's t‐test.

**Fig. S6.** Gdpd2 deficiency has no effect on BALF cells. Bronchoalveolar lavage fluid (BALF) harvested from ovalbumin (OVA)‐challenged wild‐type (WT) or OVA‐challenged Gdpd2 KO (X^KO^Y) male mice (n = 7:7). Inflammatory cells were quantified using Hema 3 staining. Results are represented by mean ± SD.

**Table S1.** The antibodies for flow cytometry.

**Table S2.** The composition of basic culture medium.

**Table S3.** The composition of stromal‐free medium (SFM).

**Table S4.** The antibodies for immunofluorescence staining.

**Table S5.** Reagents and resources.

**Table S6.** Sequences of primers for quantitative PCR.

## Data Availability

The data that support the findings of this study are available from the corresponding author upon reasonable request. The scRNA‐Seq data were obtained from the GEO database (https://www.ncbi.nlm.nih.gov/geo/), accession number: GSE203079.
