# Supplementary material for: Nitric oxide hinders club cell proliferation through Gdpd2 during allergic airway inflammation
Source: FEBS Open Bio. 2023 May 3;13(6):1041–55. doi: 10.1002/2211-5463.13617 (PMC10240343; doi:10.1002/2211-5463.13617)
Supplement: Supplementary file 1 — Fig. S1. Nitric oxide inhibits club cell proliferation in stromal‐feed organoid cultures. (A) Representative images of club cell organoid cultures (co‐cultured with mouse lung fibroblast cell line (MLg)) in the presence of 25 μM diethylamine NONOate (DEA NONOate) at day 8 after plating (n = 5:5). Scale bar: 500 μm. (B, C) Diameter and CFEs of club cell colonies from the DEA NONOate group under the conditions described in (A) (n = 5:5). (D) Numbers of MLg in control and nitric oxide (NO)‐treated groups (n = 6:6). (E, F, G) qPCR analysis of Fgf7 (E), Fgf10 (F), and Hgf (G) expressions (relative to β‐actin) of MLg, cultured in a 100‐mm petri dish for 72 h in the presence of DEA NONOate (25 μM) (n = 6:6). Results are represented by mean ± SD, ****p < 0.0001; as determined by Student's t‐test. [file FEB4-13-1041-s010.pptx]

## Slide 1
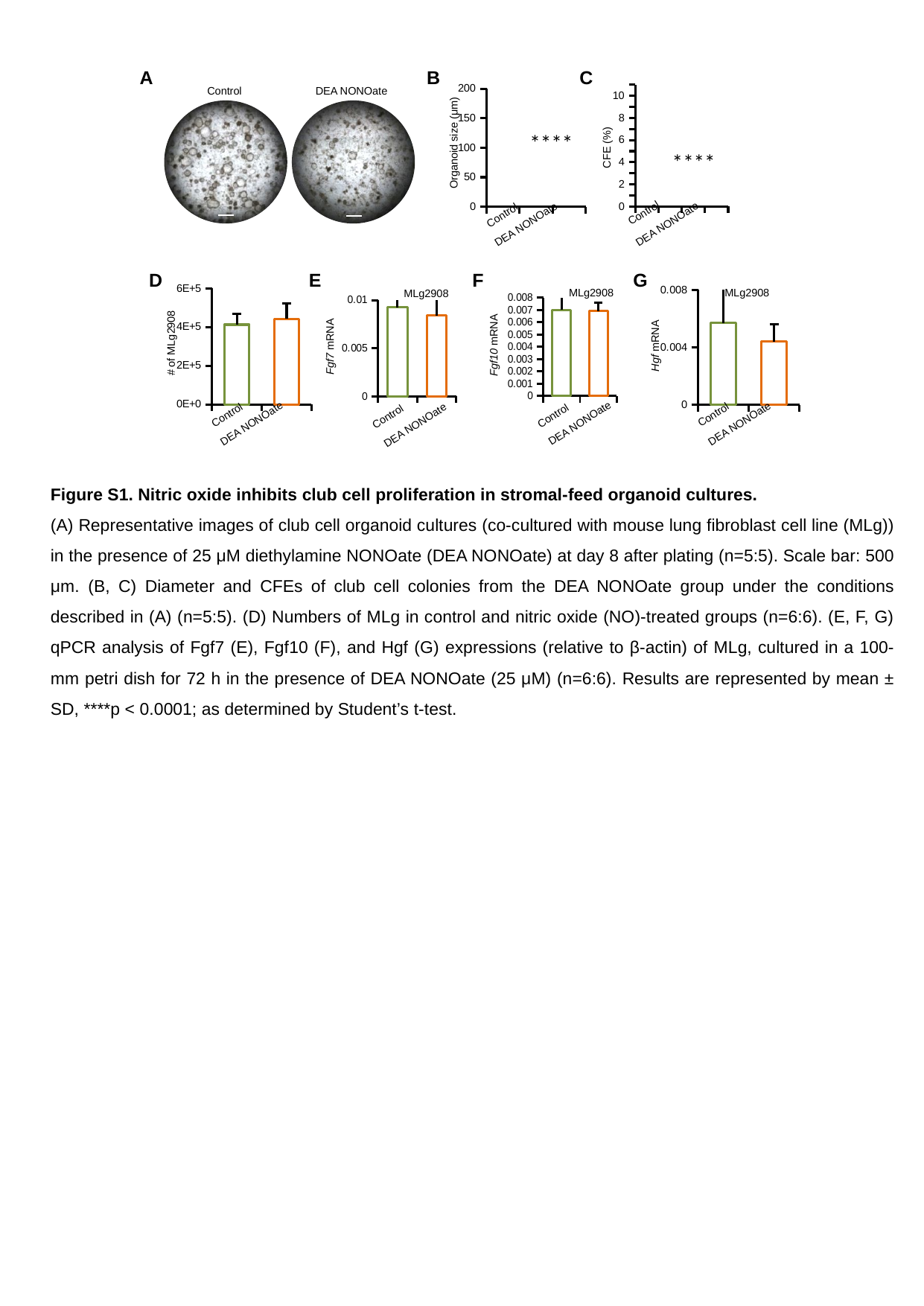

A
B
C
### Chart
| Category | |
|---|---|
| Control | 159.2024195057488 |
| DEA NONOate 25μM | 90.76998576371118 |****
Organoid size (μm)
Control
DEA NONOate
### Chart
| Category | |
|---|---|
| Control | 2.588 |
| DEA NONOate 25μM | 0.504 |CFE (%)
****
Control
DEA NONOate
Control
DEA NONOate
D
E
F
G
### Chart
| Category | |
|---|---|
| CTL | 412500.0 |
| DEA NONOate 25μM | 441666.6666666667 |# of MLg2908
Control
DEA NONOate
### Chart
| Category | |
|---|---|
| CTL | 0.005696792382277111 |
| DEA NONOate 25μM | 0.004400547201412221 |Hgf mRNA
Control
DEA NONOate
MLg2908
MLg2908
### Chart
| Category | |
|---|---|
| CTL | 0.006994959629747127 |
| DEA NONOate 25μM | 0.006906116417940644 |Fgf10 mRNA
Control
DEA NONOate
MLg2908
### Chart
| Category | |
|---|---|
| CTL | 0.009244541861251976 |
| DEA NONOate 25μM | 0.008422755344968032 |Fgf7 mRNA
Control
DEA NONOate
Figure S1. Nitric oxide inhibits club cell proliferation in stromal-feed organoid cultures.
(A) Representative images of club cell organoid cultures (co-cultured with mouse lung fibroblast cell line (MLg)) in the presence of 25 μM diethylamine NONOate (DEA NONOate) at day 8 after plating (n=5:5). Scale bar: 500 μm. (B, C) Diameter and CFEs of club cell colonies from the DEA NONOate group under the conditions described in (A) (n=5:5). (D) Numbers of MLg in control and nitric oxide (NO)-treated groups (n=6:6). (E, F, G) qPCR analysis of Fgf7 (E), Fgf10 (F), and Hgf (G) expressions (relative to β-actin) of MLg, cultured in a 100-mm petri dish for 72 h in the presence of DEA NONOate (25 μM) (n=6:6). Results are represented by mean ± SD, ****p < 0.0001; as determined by Student’s t-test.
