# Supplementary material for: Nitric oxide hinders club cell proliferation through Gdpd2 during allergic airway inflammation
Source: FEBS Open Bio. 2023 May 3;13(6):1041–55. doi: 10.1002/2211-5463.13617 (PMC10240343; doi:10.1002/2211-5463.13617)
Supplement: Supplementary file 2 — Fig. S2. Enrichment analysis of transcription factor from bulk RNA‐seq. Main enriched transcription factors of the differentially expressed genes identified by iRegulon. The normalized enrichment scores (NES) and the number of target genes are indicated. [file FEB4-13-1041-s009.pptx]

## Slide 1
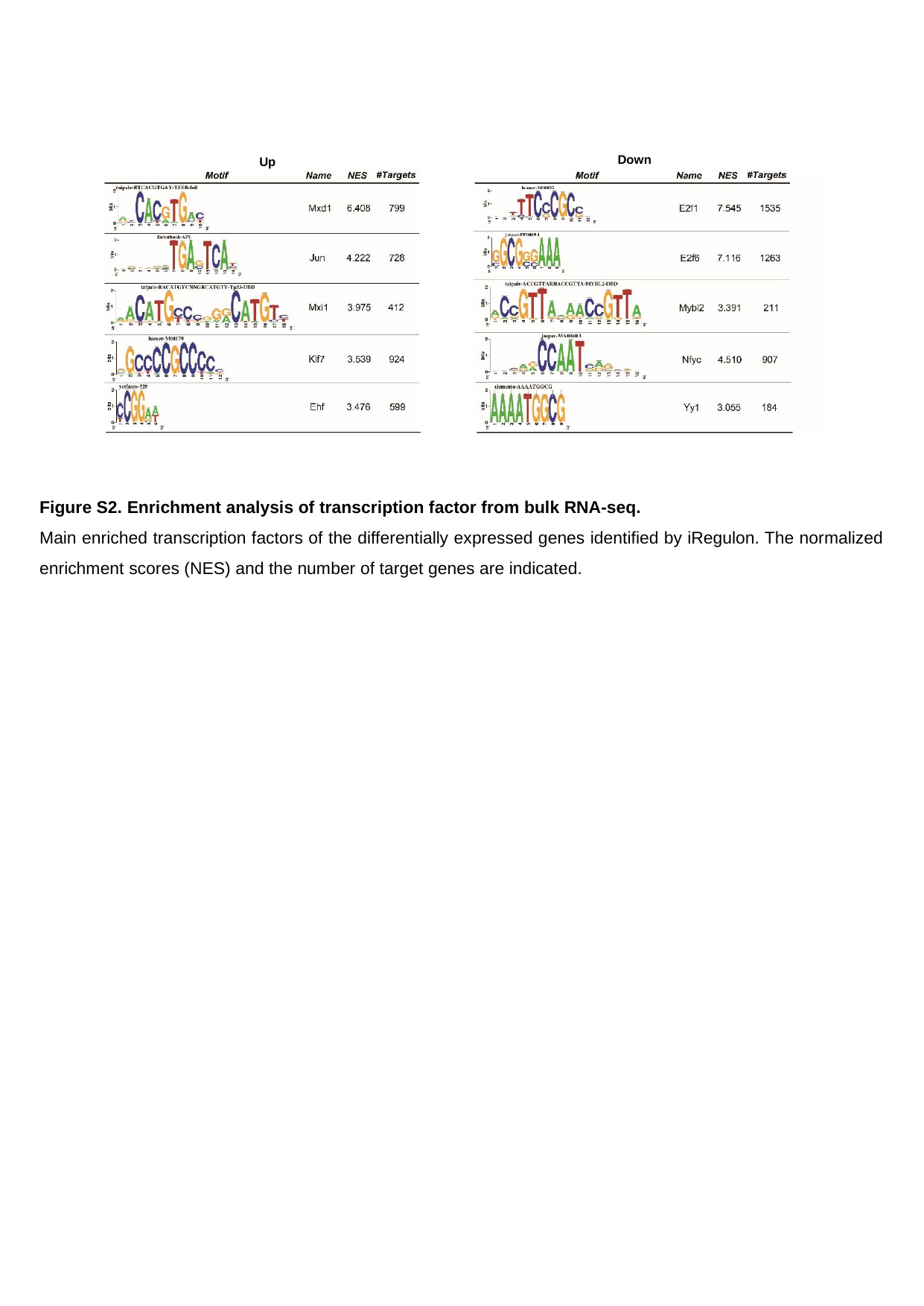

Down
Up
Figure S2. Enrichment analysis of transcription factor from bulk RNA-seq.
Main enriched transcription factors of the differentially expressed genes identified by iRegulon. The normalized enrichment scores (NES) and the number of target genes are indicated.
