# Supplementary material for: Nitric oxide hinders club cell proliferation through Gdpd2 during allergic airway inflammation
Source: FEBS Open Bio. 2023 May 3;13(6):1041–55. doi: 10.1002/2211-5463.13617 (PMC10240343; doi:10.1002/2211-5463.13617)
Supplement: Supplementary file 3 — Fig. S3. Identification of club subsets during OVA‐induced allergic inflammation. (A) Dot map depicting specific marker genes for each cell subtype. (B) scRNA‐Seq analysis of club and goblet cells percentage in total airway cells. Airway cells consist of club cell, goblet cell, basal cell and ciliated cell. (C) Specific marker genes for proliferative, apoptotic, and quiescent club cells, and goblet cells. (D) uMAP plots of club cells subsets and goblet cells. (E) Sample origin of club and goblet cells. Blue: PBS group. Red: ovalbumin (OVA) group. (F) Expression of specific marker genes of proliferative club, apoptotic club, quiescent club, and goblet cells. (G) Plots of proliferative and apoptotic scores (AUCell score and Module score) for three club cell subtypes using corresponding cell type markers. The AUCell scores are represented by mean ± SD; The Module scores are represented by median. Differences among the groups were analyzed by the two‐sided Wilcoxon rank‐sum test by wilcox.test function of the stats package. [file FEB4-13-1041-s007.pptx]

## Slide 1
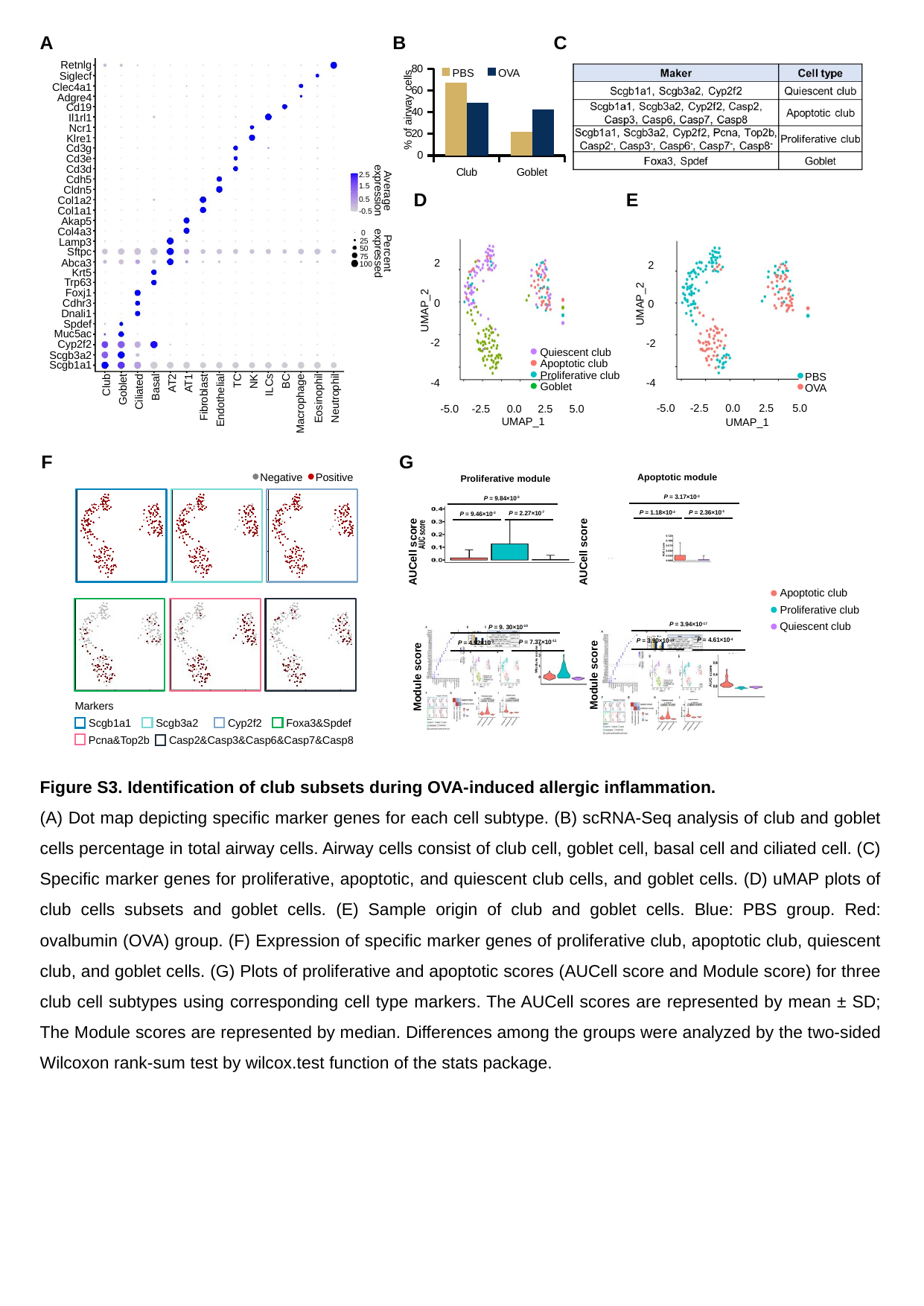

A
B
C
Retnlg
Siglecf
Clec4a1
Adgre4
Cd19
Il1rl1
Ncr1
Klre1
Cd3g
Cd3e
Cd3d
2.5
Cdh5
Average
expression
1.5
Cldn5
Col1a2
0.5
Col1a1
-0.5
Akap5
Col4a3
0
Lamp3
25
50
Percent
expressed
Sftpc
75
Abca3
100
Krt5
Trp63
Foxj1
Cdhr3
Dnali1
Spdef
Muc5ac
Cyp2f2
Scgb3a2
Scgb1a1
Club
Goblet
Ciliated
Basal
AT2
AT1
Fibroblast
Endothelial
TC
NK
ILCs
BC
Eosinophil
Neutrophil
Macrophage
### Chart
| Category | PBS | OVA |
|---|---|---|
| Club | 67.17557251908397 | 48.64864864864865 |
| Goblet | 22.137404580152673 | 42.34234234234234 |PBS
OVA
% of airway cells
D
E
2
0
UMAP_2
-2
Quiescent club
Apoptotic club
Proliferative club
Goblet
-4
-5.0
-2.5
0.0
2.5
5.0
UMAP_1
2
0
UMAP_2
-2
PBS
OVA
-4
-2.5
0.0
2.5
5.0
-5.0
UMAP_1
F
G
Apoptotic module
Proliferative module
P = 3.17×10^-3^
P = 2.36×10^-1^
P = 1.18×10^-2^
P = 9.84×10^-3^
P = 2.27×10^-7^
P = 9.46×10^-3^
AUCell score
AUCell score
Apoptotic club
Proliferative club
Quiescent club
P = 3.94×10^-17^
P = 9. 30×10-10
P = 4.61×10^-4^
P = 3.90×10^-15^
P = 7.37×10^-11^
P = 4.92×10^-3^
Module score
Module score
Negative
Positive
Markers
Scgb1a1
Scgb3a2
Cyp2f2
Foxa3&Spdef
Pcna&Top2b
Casp2&Casp3&Casp6&Casp7&Casp8
Figure S3. Identification of club subsets during OVA-induced allergic inflammation.
(A) Dot map depicting specific marker genes for each cell subtype. (B) scRNA-Seq analysis of club and goblet cells percentage in total airway cells. Airway cells consist of club cell, goblet cell, basal cell and ciliated cell. (C) Specific marker genes for proliferative, apoptotic, and quiescent club cells, and goblet cells. (D) uMAP plots of club cells subsets and goblet cells. (E) Sample origin of club and goblet cells. Blue: PBS group. Red: ovalbumin (OVA) group. (F) Expression of specific marker genes of proliferative club, apoptotic club, quiescent club, and goblet cells. (G) Plots of proliferative and apoptotic scores (AUCell score and Module score) for three club cell subtypes using corresponding cell type markers. The AUCell scores are represented by mean ± SD; The Module scores are represented by median. Differences among the groups were analyzed by the two-sided Wilcoxon rank-sum test by wilcox.test function of the stats package.
