# Supplementary material for: Nitric oxide hinders club cell proliferation through Gdpd2 during allergic airway inflammation
Source: FEBS Open Bio. 2023 May 3;13(6):1041–55. doi: 10.1002/2211-5463.13617 (PMC10240343; doi:10.1002/2211-5463.13617)
Supplement: Supplementary file 4 — Fig. S4. Altered subsets of club cells during OVA‐induced allergic inflammation. (A) Pseudotime developmental trajectory analysis from Monocle2 depicting relations between proliferative club, apoptotic club, quiescent club, and goblet cells. (B) Pseudotime labelling of goblet cells and three club cells subsets. (C) Club cells subtypes percentage in total airway cells. (D) Single‐cell RNA seq analysis of Gdpd2 expression in each club cells subset. (E, F) Single‐cell RNA seq analysis of Gdpd2 expression in apoptotic (E) or proliferative (F) club cells. Differences were analyzed by the two‐sided Wilcoxon rank‐sum test based on the FindMarkers function of the Seurat package, p < 0.05 were considered to be statistically significant. (G) Expression heatmap showing top 50 genes with the lowest q value along the cell differentiation. (H) Top 20 pathway enrichment results of genes altered in differentiation process. [file FEB4-13-1041-s002.pptx]

## Slide 1
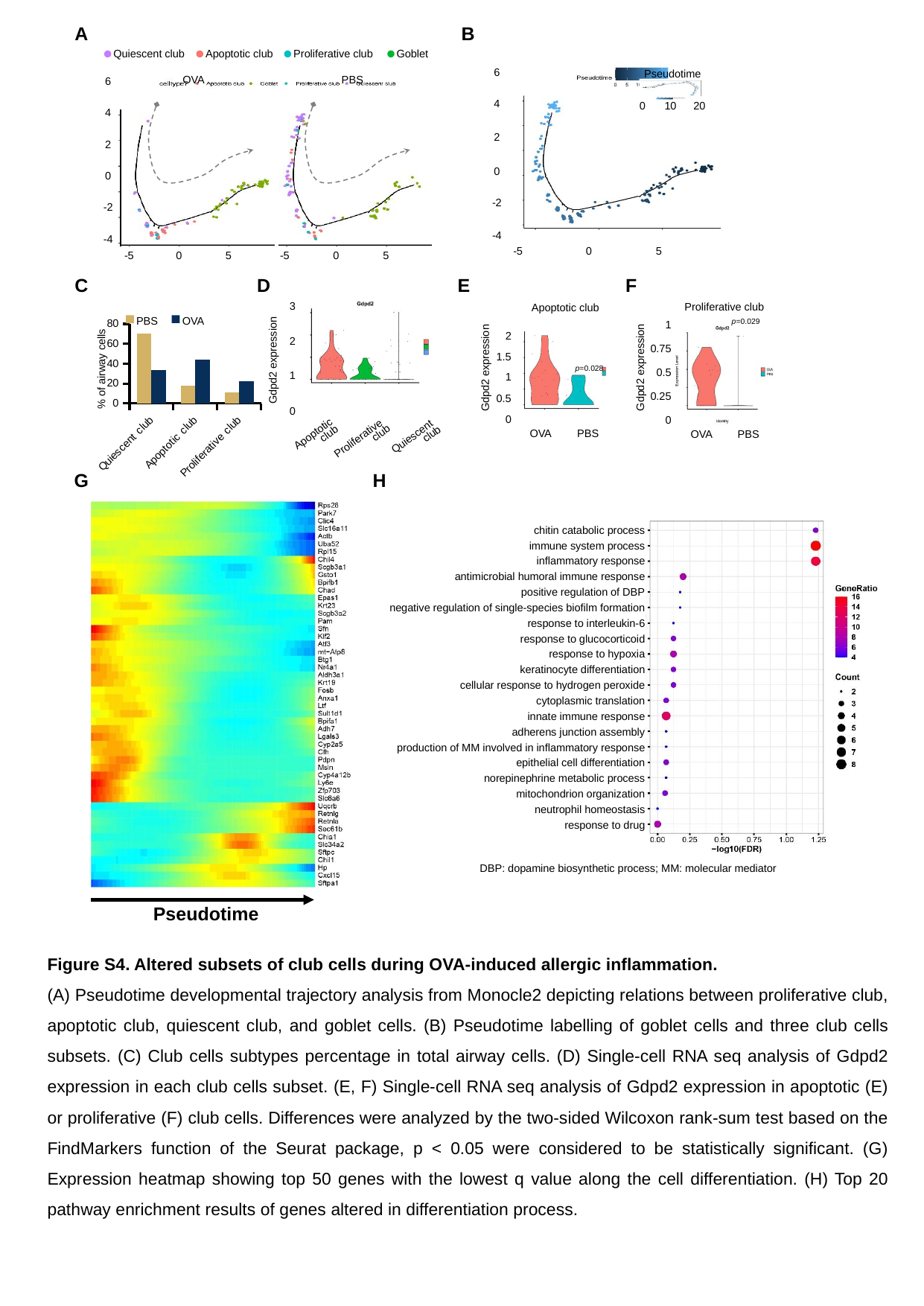

A
B
Quiescent club
Apoptotic club
Proliferative club
Goblet
PBS
OVA
6
4
2
0
-2
-4
5
-5
0
-5
0
5
6
Pseudotime
4
0
10
20
2
0
-2
-4
-5
0
5
C
D
E
F
3
2
Gdpd2 expression
1
0
Apoptotic
club
Quiescent
club
Proliferative
club
Proliferative club
p=0.029
1
0.75
0.5
0.25
0
PBS
OVA
Gdpd2 expression
Apoptotic club
2
1.5
Gdpd2 expression
p=0.028
1
0.5
0
PBS
OVA
PBS
OVA
### Chart
| Category | PBS | OVA |
|---|---|---|
| Quiescent club | 70.45454545454545 | 33.33333333333333 |
| Apoptotic club | 18.181818181818183 | 44.44444444444444 |
| Proliferative club | 11.363636363636363 | 22.22222222222222 |% of airway cells
G
H
Pseudotime
chitin catabolic process
immune system process
inflammatory response
antimicrobial humoral immune response
positive regulation of DBP
negative regulation of single-species biofilm formation
response to interleukin-6
response to glucocorticoid
response to hypoxia
keratinocyte differentiation
cellular response to hydrogen peroxide
cytoplasmic translation
innate immune response
adherens junction assembly
production of MM involved in inflammatory response
epithelial cell differentiation
norepinephrine metabolic process
mitochondrion organization
neutrophil homeostasis
response to drug
DBP: dopamine biosynthetic process; MM: molecular mediator
Figure S4. Altered subsets of club cells during OVA-induced allergic inflammation.
(A) Pseudotime developmental trajectory analysis from Monocle2 depicting relations between proliferative club, apoptotic club, quiescent club, and goblet cells. (B) Pseudotime labelling of goblet cells and three club cells subsets. (C) Club cells subtypes percentage in total airway cells. (D) Single-cell RNA seq analysis of Gdpd2 expression in each club cells subset. (E, F) Single-cell RNA seq analysis of Gdpd2 expression in apoptotic (E) or proliferative (F) club cells. Differences were analyzed by the two-sided Wilcoxon rank-sum test based on the FindMarkers function of the Seurat package, p < 0.05 were considered to be statistically significant. (G) Expression heatmap showing top 50 genes with the lowest q value along the cell differentiation. (H) Top 20 pathway enrichment results of genes altered in differentiation process.
