# Supplementary material for: Nitric oxide hinders club cell proliferation through Gdpd2 during allergic airway inflammation
Source: FEBS Open Bio. 2023 May 3;13(6):1041–55. doi: 10.1002/2211-5463.13617 (PMC10240343; doi:10.1002/2211-5463.13617)
Supplement: Supplementary file 5 — Fig. S5. Gdpd2 deficiency inhibits club cell proliferation in vitro. (A) Representative images of club cell organoid cultures (stromal‐free system) from wide‐type mice or Gdpd2 KO (XKOXWT) mice (n = 5:5), at day 8 after plating. Scale bar: 500 μm. (B, C) Diameter and CFEs of club cell colonies under the conditions described in (A) (n = 5:5). (D) Representative images of club cell organoid cultures (stromal‐free system) from wide‐type mice or Gdpd2 KO (XKOY) mice (n = 5:5), at day 8 after plating. Scale bar: 500 μm. (E, F) Diameter and CFEs of club cell colonies under the conditions described in (C) (n = 5:5). Results are represented by mean ± SD, *p < 0.05, ***p < 0.001, ****p < 0.0001; as determined by Student's t‐test. [file FEB4-13-1041-s005.pptx]

## Slide 1
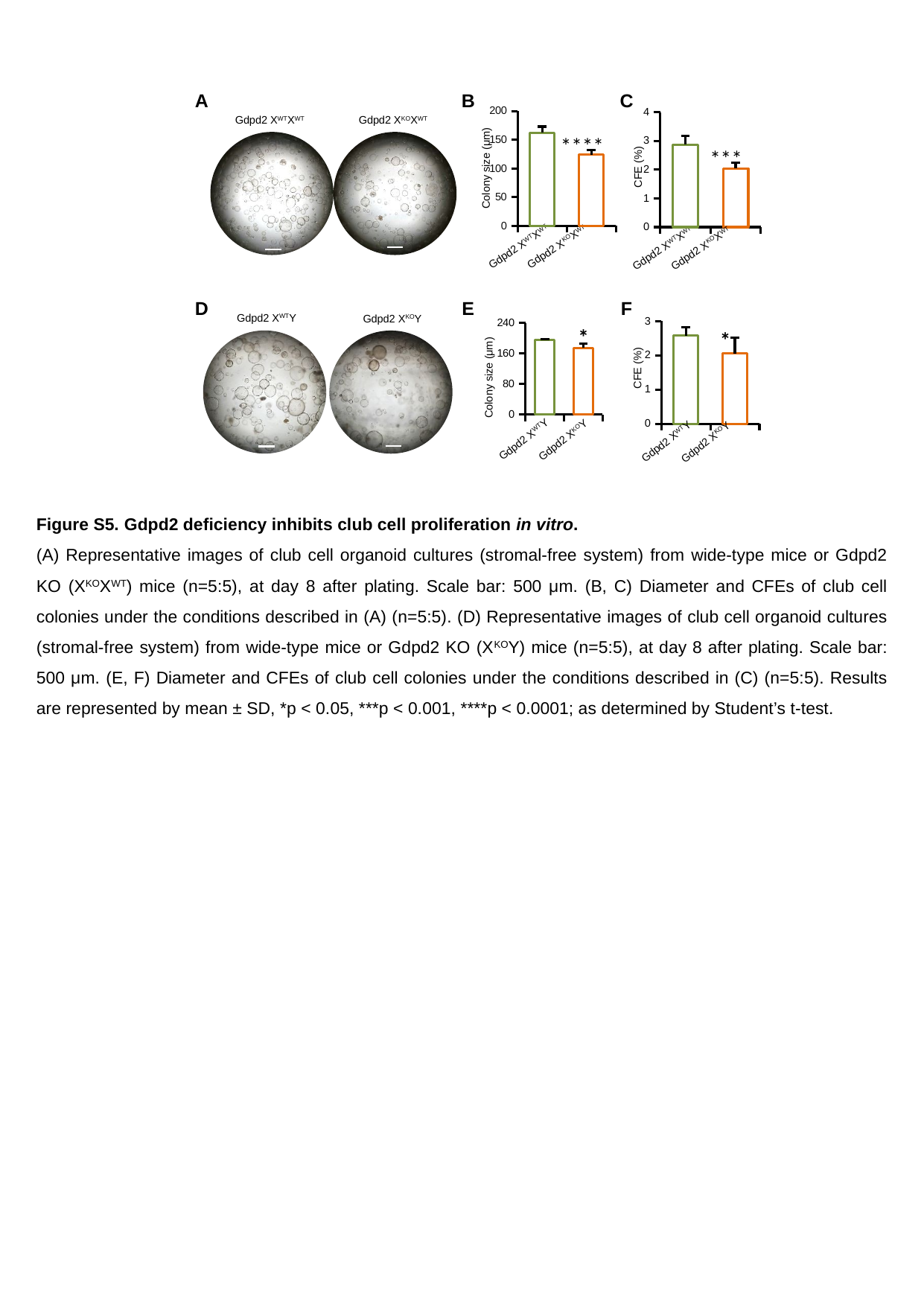

A
B
C
### Chart
| Category | AVE |
|---|---|
| CTL | 161.92248425381507 |
| KO | 124.05880390440362 |Colony size (μm)
****
Gdpd2 XKOXWT
Gdpd2 XWTXWT
### Chart
| Category | AVE |
|---|---|
| CTL | 2.868 |
| KO | 2.04 |***
CFE (%)
Gdpd2 XKOXWT
Gdpd2 XWTXWT
Gdpd2 XWTXWT
Gdpd2 XKOXWT
D
E
F
### Chart
| Category | |
|---|---|
| CTL | 196.08279065168523 |
| Gdpd2 KO | 174.6451378693994 |Colony size (μm)
*
Gdpd2 XWTY
Gdpd2 XKOY
Gdpd2 XWTY
Gdpd2 XKOY
### Chart
| Category | |
|---|---|
| CTL | 2.572 |
| DEA NONOate | 2.0500000000000003 |*
Gdpd2 XWTY
Gdpd2 XKOY
CFE (%)
Figure S5. Gdpd2 deficiency inhibits club cell proliferation in vitro.
(A) Representative images of club cell organoid cultures (stromal-free system) from wide-type mice or Gdpd2 KO (XKOXWT) mice (n=5:5), at day 8 after plating. Scale bar: 500 μm. (B, C) Diameter and CFEs of club cell colonies under the conditions described in (A) (n=5:5). (D) Representative images of club cell organoid cultures (stromal-free system) from wide-type mice or Gdpd2 KO (XKOY) mice (n=5:5), at day 8 after plating. Scale bar: 500 μm. (E, F) Diameter and CFEs of club cell colonies under the conditions described in (C) (n=5:5). Results are represented by mean ± SD, *p < 0.05, ***p < 0.001, ****p < 0.0001; as determined by Student’s t-test.
