# Supplementary material for: Nitric oxide hinders club cell proliferation through Gdpd2 during allergic airway inflammation
Source: FEBS Open Bio. 2023 May 3;13(6):1041–55. doi: 10.1002/2211-5463.13617 (PMC10240343; doi:10.1002/2211-5463.13617)
Supplement: Supplementary file 6 — Fig. S6. Gdpd2 deficiency has no effect on BALF cells. Bronchoalveolar lavage fluid (BALF) harvested from ovalbumin (OVA)‐challenged wild‐type (WT) or OVA‐challenged Gdpd2 KO (XKOY) male mice (n = 7:7). Inflammatory cells were quantified using Hema 3 staining. Results are represented by mean ± SD. [file FEB4-13-1041-s012.pptx]

## Slide 1
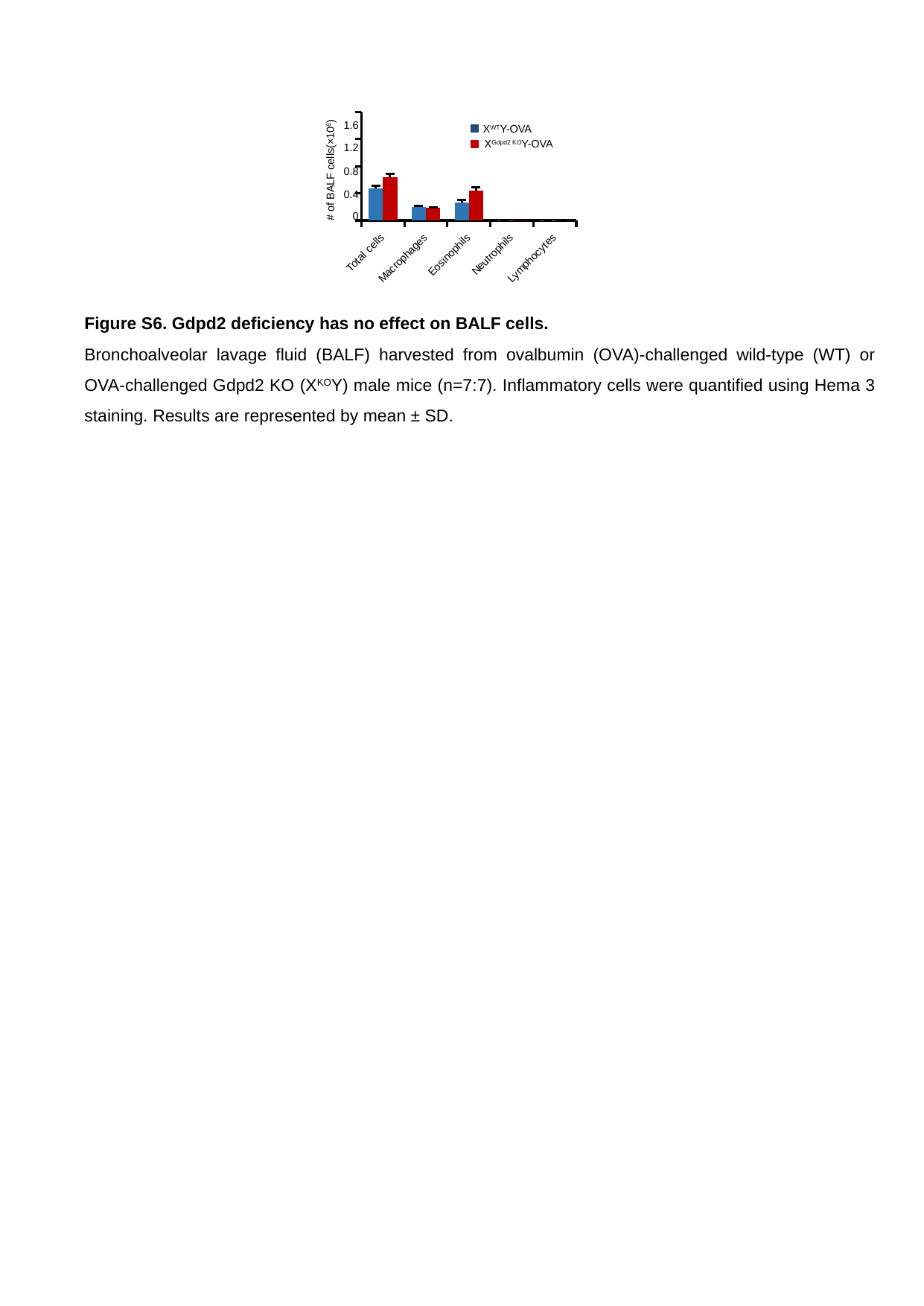

### Chart
| Category | OVA XWT/Y | OVA XKO/Y |
|---|---|---|
| Total cells | 472404.0689960195 | 636649.4282737678 |
| Macrophages | 200368.91994692615 | 186093.75480204154 |
| Eosinophils | 266510.8076072534 | 442169.6550749116 |
| Neutrophils | 1407.4338788146836 | 2811.1055711457716 |
| Lymphocytes | 4116.90756302521 | 5574.912825668784 |1.6
XWTY-OVA
XGdpd2 KOY-OVA
1.2
0.8
0.4
0
# of BALF cells(×106)
Figure S6. Gdpd2 deficiency has no effect on BALF cells.
Bronchoalveolar lavage fluid (BALF) harvested from ovalbumin (OVA)-challenged wild-type (WT) or OVA-challenged Gdpd2 KO (XKOY) male mice (n=7:7). Inflammatory cells were quantified using Hema 3 staining. Results are represented by mean ± SD.
