# Supplementary material for: Nitric oxide hinders club cell proliferation through Gdpd2 during allergic airway inflammation
Source: FEBS Open Bio. 2023 May 3;13(6):1041–55. doi: 10.1002/2211-5463.13617 (PMC10240343; doi:10.1002/2211-5463.13617)
Supplement: Supplementary file 7 — Table S1. The antibodies for flow cytometry. [file FEB4-13-1041-s011.docx]

**Table S1.** **The antibodies for flow cytometry**

| Antibodies | Source | Identifier |
| --- | --- | --- |
| Anti-Mouse CD24 PE | eBioscience | Cat#: 12-0242-83 |
| Anti-Mouse CD45 Biotin | eBioscience | Cat#: 13-0451-85 |
| Anti-Mouse CD34 Biotin | eBioscience | Cat#: 13-0341-85 |
| Anti-Mouse CD31 Biotin | eBioscience | Cat#: 13-0311-85 |
| Anti-Mouse Ly-6A/E (Sca-1) APC | eBioscience | Cat#: 17-5981-81 |
| Anti-Mouse CD11c APC | eBioscience | Cat#: 17-0114-82 |
| PE/Cy7 anti-mouse CD326 (Ep-CAM) | eBioscience | Cat#: 118216 |
| 7-AAD Viability Staining Solution | eBioscience | Cat#: 00-6993-50 |
| Streptavidin APC-eFluor 780 | eBioscience | Cat#: 47-4317-82 |
