# Supplementary material for: Nitric oxide hinders club cell proliferation through Gdpd2 during allergic airway inflammation
Source: FEBS Open Bio. 2023 May 3;13(6):1041–55. doi: 10.1002/2211-5463.13617 (PMC10240343; doi:10.1002/2211-5463.13617)
Supplement: Supplementary file 8 — Table S2. The composition of basic culture medium. [file FEB4-13-1041-s006.docx]

**Table S2.** **The composition of basic culture medium**

| Component | Concentration | Treatment period |  |
| --- | --- | --- | --- |
| Base medium | DMEM/F12 | | |
| FBS | 10% | Day 0-Day 8 |  |
| PS | 1% | Day 0-Day 8 |  |
| ITS | 1% | Day 0-Day 8 |  |
| Y27632 | 1 μM | Day 0-Day 4 |  |
| SB431542 | 100 nM | Day 0-Day 8 |  |
