# Supplementary material for: Nitric oxide hinders club cell proliferation through Gdpd2 during allergic airway inflammation
Source: FEBS Open Bio. 2023 May 3;13(6):1041–55. doi: 10.1002/2211-5463.13617 (PMC10240343; doi:10.1002/2211-5463.13617)
Supplement: Supplementary file 9 — Table S3. The composition of stromal‐free medium (SFM). [file FEB4-13-1041-s004.docx]

**Table S3.** **The composition of stromal-free medium (SFM)**

| Component | Concentration | Treatment period |  |
| --- | --- | --- | --- |
| Base medium | DMEM/F12 | | |
| FBS | 10% | Day 0-Day 8 |  |
| PS | 1% | Day 0-Day 8 |  |
| ITS | 1% | Day 0-Day 8 |  |
| HEPES | 1% | Day 0-Day 8 |  |
| B27S | 2% | Day 0-Day 8 |  |
| FGF-7 | 25 ng/mL | Day 0-Day 8 |  |
| CHIR99021 | 3 μM | Day 0-Day 8 |  |
| FGF-10 | 10 ng/mL | Day 0-Day 8 |  |
| Y27632 | 1 μM | Day 0-Day 4 |  |
| Noggin | 100 ng/mL | Day 0-Day 8 |  |
| R-spondin 1 | 500 ng/mL | Day 0-Day 8 |  |
| SB202190 | 1 μM | Day 0-Day 8 |  |
| SB431542 | 100 nM | Day 0-Day 8 |  |
