# Supplementary material for: Nitric oxide hinders club cell proliferation through Gdpd2 during allergic airway inflammation
Source: FEBS Open Bio. 2023 May 3;13(6):1041–55. doi: 10.1002/2211-5463.13617 (PMC10240343; doi:10.1002/2211-5463.13617)
Supplement: Supplementary file 10 — Table S4. The antibodies for immunofluorescence staining. [file FEB4-13-1041-s003.docx]

**Table S4.** **The antibodies for immunofluorescence staining**

| Antibodies | Source | Identifier |
| --- | --- | --- |
| Anti-CCSP (CC10) | Seven Hills Bioreagents | Cat#: WRAB-3950 |
| Anti-Clca1 | abcam | Cat#: Ab180851 |
| Anti-ki67 | Invitrogen | Cat#: 14-5698-82 |
| Anti-CYP2F2 | Santa Cruz | Cat#: sc-374540 |
| DAPI | Roche | Cat#: 10236276001 |
| Donkey anti-Mouse IgG, Alexa Fluor 594 | Invitrogen | Cat#: A21203 |
| Donkey anti-Rabbit IgG, Alexa Fluor 488 | Invitrogen | Cat#: A21206 |
| Goat anti-Rat IgG, Alexa Fluor 488 | Invitrogen | Cat#: A11006 |
| Donkey anti-Rabbit IgG, Alexa Fluor 594 | Invitrogen | Cat#: A21207 |
