# Supplementary material for: Nitric oxide hinders club cell proliferation through Gdpd2 during allergic airway inflammation
Source: FEBS Open Bio. 2023 May 3;13(6):1041–55. doi: 10.1002/2211-5463.13617 (PMC10240343; doi:10.1002/2211-5463.13617)
Supplement: Supplementary file 11 — Table S5. Reagents and resources. [file FEB4-13-1041-s001.docx]

**Table S5.** **Reagents and resources**

| **REAGENT or RESOURCE** | **SOURCE** | **IDENTIFIER** |
| --- | --- | --- |
| Chemicals, Peptides, and Recombinant Proteins | | |
| Matrigel^TM^ Matrix | BD Pharmingen | Cat#: 356231 |
| DMEM-F12 | Corning | Cat#: 16-405-CVR |
| FBS | Gibco | Cat#: 16000-044 |
| Penicillin-streptomycin | Gibco | Cat#: 15140-122 |
| ITS | Sigma-Aldrich | Cat#: I3146 |
| HEPES | Sigma-Aldrich | Cat#: H0087 |
| B27 Supplement | Gibco | Cat#: 17504-044 |
| Human KGF (FGF-7) | PeproTech | Cat#: 100-19-10 |
| Human FGF10/KGF2 Protein | SinoBiological | Cat#: 10573-HNAE |
| Noggin Protein, Human | MCE | Cat#: HY-P70558 |
| RSPO1/R-spondin-1 Protein, Human | MCE | Cat#: HY-P72784 |
| Laduviglusib (CHIR99021) | MCE | Cat#: HY-10182 |
| Y27632 | Sigma-Aldrich | Cat#: Y0503 |
| SB431542 | Sigma-Aldrich | Cat#: S4317-5MG |
| SB202190 | MCE | Cat#: HY-10295 |
| Ham's F12 | Corning | Cat#: 10-080-CVR |
| DeoxyribonucleaseⅠfrom bovine pancreas | Sigma-Aldrich | Cat#: DN25-5G |
| EGTA | Sigma-Aldrich | Cat#: E8145 |
| Albumin from chicken egg white (OVA) | Sigma-Aldrich | Cat#: A5503-50g |
| TRIzol reagent | Invitrogen | Cat#: 15596018 |
| HBSS | Solarbio | Cat#: H1025 |
| 4% paraformaldehyde | Solarbio | Cat#: P1110 |
| Triton X-100 | Sangon Biotech | Cat#: A600198-0500 |
| 5 % BSA | BOSTER | Cat#: AR0004 |
| Elastase | Worthington Biochemical Corporation | Cat#: LS002279 |
| HiScript® III RT SuperMix | Vazyme | Cat#: R323-01 |
| ChamQ Universal SYBR qPCR Master Mix | Vazyme | Cat#: Q711 |
| VECTASHIELD | Vectorlabs | Cat#: H1000 |
| Imject Alum Adjuvant | Thermo Fisher Scientific | Cat#: 77161 |
| Hema 3^TM^ | Fisherbrand | Cat#: 400937 |
| DEA NONOate | Cayman | Cat#: 82100 |
| Glycerol | Sangon Biotech | Cat#: A100854-0100 |
| D-myo-Inositol-1-phosphate | Cayman | Cat#: 10007777 |
| L-NMMA | Biyotime | Cat#: S0011 |
| Critical Commercial Assays |  |  |
| Nitric Oxide (NO) assay kit | Nanjing jiancheng Bioengineering Institute | Cat#: A012-1-2 |
| DeadEnd^TM^ Fluorometric TUNEL System | Promega | Cat#: G3250 |
| MLg cell line |  |  |
| MLg2908 mouse lung fibroblast cell line | ATCC | Cat#: CCL-206 |
