# Supplementary material for: Nitric oxide hinders club cell proliferation through Gdpd2 during allergic airway inflammation
Source: FEBS Open Bio. 2023 May 3;13(6):1041–55. doi: 10.1002/2211-5463.13617 (PMC10240343; doi:10.1002/2211-5463.13617)
Supplement: Supplementary file 12 — Table S6. Sequences of primers for quantitative PCR. [file FEB4-13-1041-s008.docx]

**Table S6.** **Sequences of primers for quantitative PCR**

| Genes | Forward primer | Reverse primer |
| --- | --- | --- |
| *β-actin* | 5’-GGCCAACCGTGAAAAGATGA-3’ | 5’-CAGCCTGGATGGCTACGTACA-3’ |
| *E-cadherin* | 5’-CTGCTGCTCCTACTGTTTCTAC-3’ | 5’-TCTTCTTCTCCACCTCCTTCT-3’ |
| *Gdpd2* | 5’-CTGCTGCTCCTCATTGTACTT-3’ | 5’-CCTGCAGTGACAGTCGTAAA-3’ |
| *Hgf* | 5’-CCTGGTGTTTCACAAGCAATC-3’ | 5’-CATGGGACCTCTGTAGCTTTC-3’ |
| *Fgf7* | 5’-GAGCGACACACCAGAAGTTAT-3’ | 5’-CCTTTCACTTTGCCTCGTTTG-3’ |
| *Fgf10* | 5’-CCGTACAGTGTCCTGGAGATAA-3’ | 5’-CCCTTCTTGTTCATGGCTAAGT-3’ |
